# Usability-Focused Development and Usage of NeoTree-Beta, an App for Newborn Care in a Low-Resource Neonatal Unit, Malawi

**DOI:** 10.3389/fpubh.2022.793314

**Published:** 2022-04-28

**Authors:** Caroline Crehan, Msandeni Chiume, Yamikani Mgusha, Precious Dinga, Tim Hull-Bailey, Charles Normand, Yali Sassoon, Deliwe Nkhoma, Kim Greenwood, Fabiana Lorencatto, Monica Lakhanpaul, Michelle Heys

**Affiliations:** ^1^Population Policy and Practice Department, Great Ormond Street Institute of Child Health, University College London, London, United Kingdom; ^2^Paediatric Department, Kamuzu Central Hospital, Lilongwe, Malawi; ^3^Spinspire Limited, London, United Kingdom; ^4^Snowplow Analytics, London, United Kingdom; ^5^Parent and Child Health Initiative, Lilongwe, Malawi; ^6^ICAgile, London, United Kingdom; ^7^Centre for Behaviour Change, University College London, London, United Kingdom

**Keywords:** neonate, low resource, mHealth, mobile app, usability, user experience, user centred design, agile

## Abstract

**Background:**

Neonatal mortality is high in low-resource settings. NeoTree is a digital intervention for neonatal healthcare professionals (HCPs) aiming to achieve data-driven quality improvement and improved neonatal survival in low-resource hospitals. Optimising usability with end-users could help digital health interventions succeed beyond pilot stages in low-resource settings. Usability is the quality of a user's experience when interacting with an intervention, encompassing their effectiveness, efficiency, and overall satisfaction.

**Objective:**

To evaluate the usability and usage of NeoTree beta-app and conduct Agile usability-focused intervention development.

**Method:**

A real-world pilot of NeoTree beta-app was conducted over 6 months at Kamuzu Central Hospital neonatal unit, Malawi. Prior to deployment, think-aloud interviews were conducted to guide nurses through the app whilst voicing their thoughts aloud (*n* = 6). System Usability Scale (SUS) scores were collected before the implementation of NeoTree into usual clinical care and 6 months after implementation (*n* = 8 and 8). During the pilot, real-world user-feedback and user-data were gathered. Feedback notes were subjected to thematic analysis within an Agile “product backlog.” For usage, number of users, user-cadre, proportion of admissions/outcomes recorded digitally, and median app-completion times were calculated.

**Results:**

Twelve overarching usability themes generated 57 app adjustments, 39 (68%) from think aloud analysis and 18 (32%) from the real-world testing. A total of 21 usability themes/issues with corresponding app features were produced and added to the app. Six themes relating to data collection included exhaustiveness of data schema, prevention of errors, ease of progression, efficiency of data entry using shortcuts, navigation of user interface (UI), and relevancy of content. Six themes relating to the clinical care included cohesion with ward process, embedded education, locally coherent language, adaptability of user-interface to available resources, and printout design to facilitate handover. SUS scores were above average (88.1 and 89.4 at 1 and 6 months, respectively). Ninety-three different HCPs of 5 cadres, recorded 1,323 admissions and 1,197 outcomes over 6 months. NeoTree achieved 100% digital coverage of sick neonates admitted. Median completion times were 16 and 8 min for admissions and outcomes, respectively.

**Conclusions:**

This study demonstrates optimisation of a digital health app in a low-resource setting and could inform other similar usability studies apps in similar settings.

## Introduction

Usability can be defined as “the quality of a user's experience when interacting with a product, encompassing effectiveness, efficiency, and overall satisfaction of the user” (1). Usability is crucial to the success of interactive healthcare applications (2) beyond pilot stages, for efficiency, avoiding staff burnout (3), patient safety, and quality of care.

The usability of digital health interventions can be measured quantitatively and qualitatively. Quantitative measures include the System Usability Scale (SUS) created by John Brooke in 1986 (4), the industry standard for the quantitative assessment of usability (3), technologies (5), and products (6). SUS is a 10-question assessment proven reliable (7) and valid in high resource settings (8, 9). Scores of more than 68 are considered acceptable or above average. Although widely used and recognised, SUS does not explore human idiosyncrasies or delineate why particular usability issues occur during a user's experience (10).

User experience is termed as “UX” and Neilson's landscape of UX research methodologies (11) for understanding these issues better, includes scripted “think-aloud” usability studies, where an early prototype is tested in theoretical conditions before being released to the real world. Participants are asked to vocalise their thoughts and experiences while user-testing an intervention (12) generating qualitative feedback regarding the “what” but also the “why” of usability issues. This in combination with SUS has been shown to be more effective at explaining usability compared with SUS alone (2, 12). User Centred Design (UCD) and Agile are both established UX philosophies involving the user-focused iterative cycles of testing and refinement. While UCD is designer-led, centred on the end-user achieving their goals, Agile is more the remit of developers, focusing on the early and continuous delivery of working software to bring commercial value as early as possible (13). An Agile “product backlog” can structure the prioritisation of the smallest functional increments of work (“User-Stories”) within larger bodies of work (“Epics”), which can then be efficiently worked on simultaneously by different “scrum-teams.” Agile-UXD approaches are now being applied more frequently to the development of health apps for the patients' own self-management of diabetes (14), paediatric concussion (15), weight management (16), and smoking cessation (17). Reports of their use for health apps in low-resource hospital settings, where usability may be particularly important, are less common. Preliminary examples of Agile development in low resource settings include the development of a smart phone app for perinatal monitoring by Guatemalan midwives (18).

**Figure 1:**
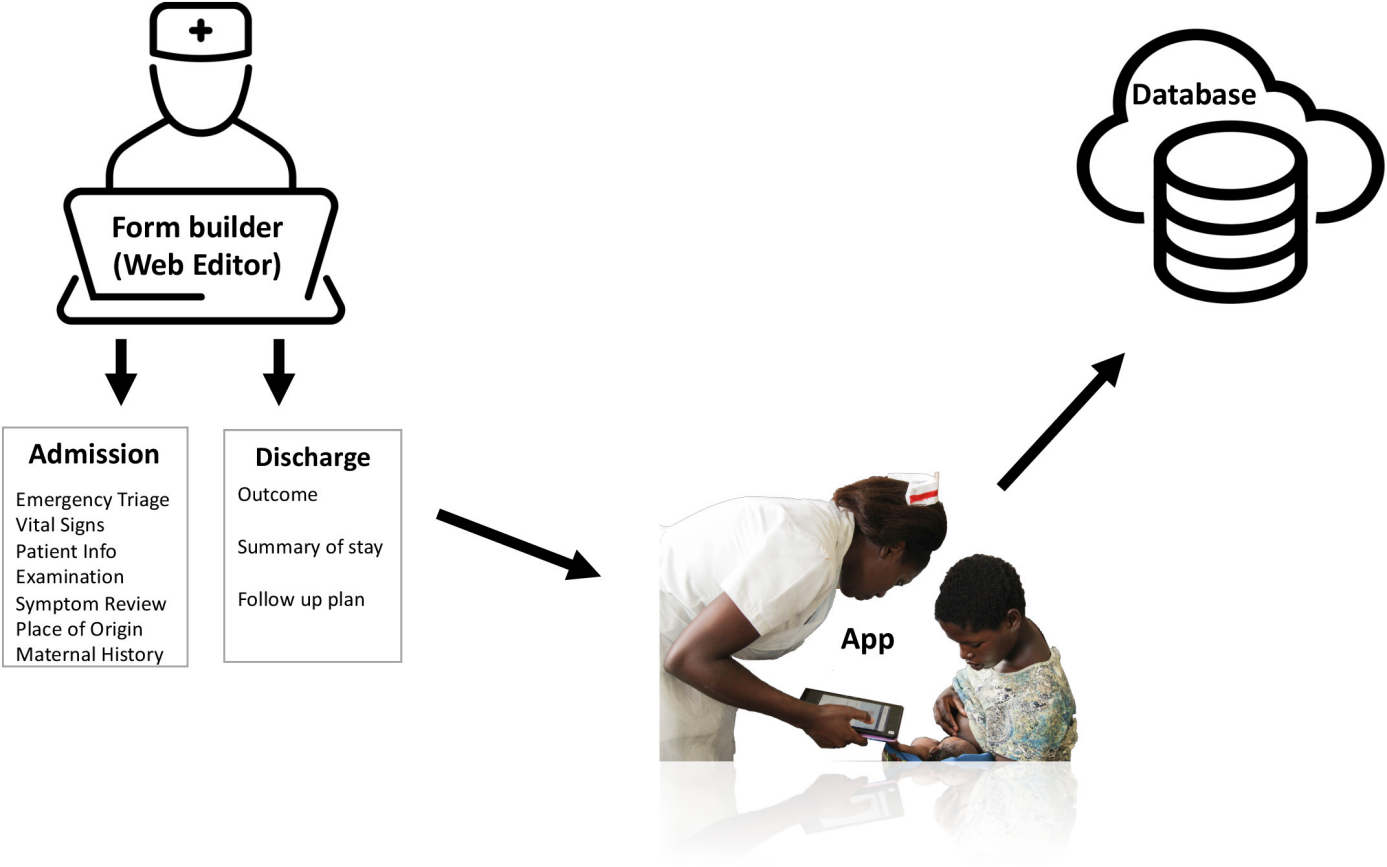
NeoTree system. Copyright 2021 Mgusha et al (19).

**Figure 2:**
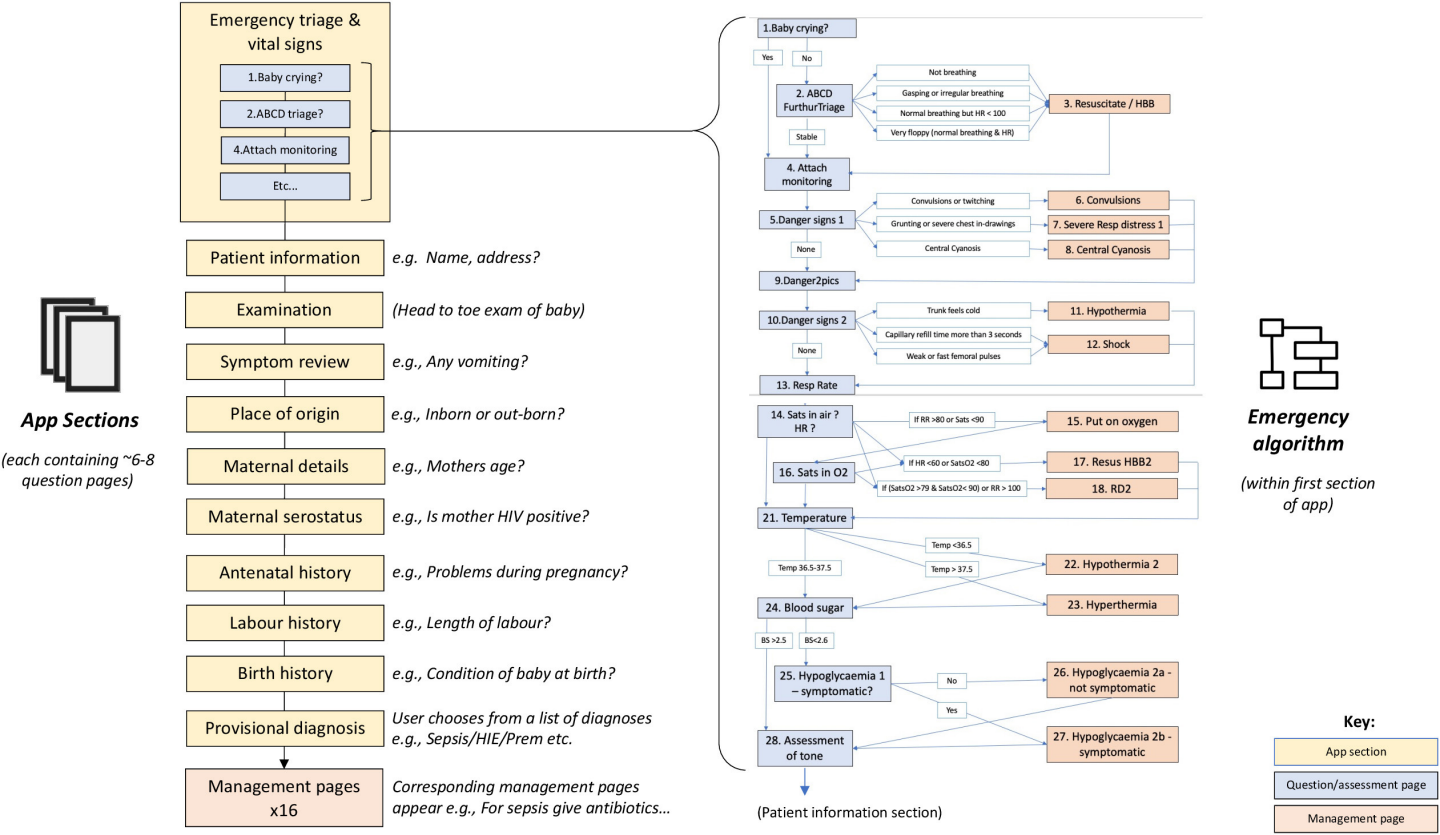
Map of NeoTree App.

**Figure 3:**
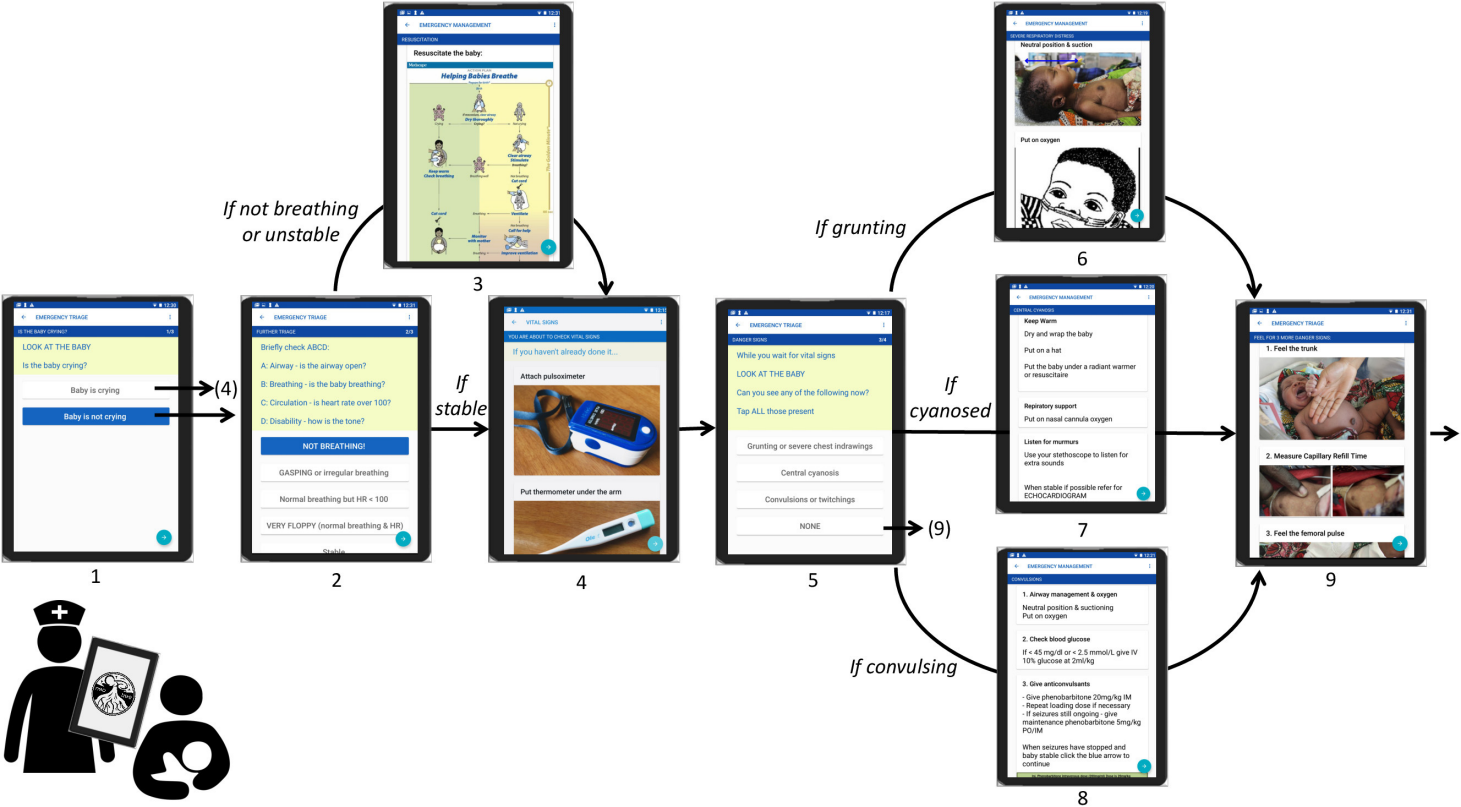
Example NeoTree screen flow (Screens 1–9; emergency triage and vital signs).

The NeoTree (Figure 1) is a recently developed, neonatal digital-health system, centred around an app which supports Health Care Professionals (HCP) working in low-resource neonatal units (20, 21). NeoTree aims to improve neonatal care and reduce newborn mortality by bringing evidence-based guidelines, algorithms, and digital data capture to the bedside. Within the NeoTree app, nurses complete digital forms, documenting admission, and discharge details for each sick newborn admitted to a neonatal unit. The app asks them to complete one field/question per page of the app. Figure 2 maps out the sections of the digital admission form that users must complete as they progress through the app, such as the embedded emergency clinical algorithm. Figure 3 shows a screen flow of the first nine pages of the app, demonstrating how a user is prompted to resuscitate a newborn who is not breathing and how to manage various newborn emergencies should they occur during admission. In this way, as the user completes the app, they receive decision support, prompts, and management support on how to manage a sick neonate according to best practise and guidance. Table 1 summarises the functions, aim, and goal of NeoTree to date.

**Table 1:** Neotree functions and purpose overview.

**Functions**	**Description of function**	**Aim**	**Goal**
1. Electronic Medical Record (EMR)	Digital data capture at admission, discharge/outcome, and lab data, facilitated via online editor platform.	Improve newborn care	Reduce newborn morbidity and mortality
2. Clinical decision support (CDS)	Facilitated through algorithmic support in emergencies (digital implementation of evidence-based guidelines)		
3. Digital guideline	Clinical management support facilitated through management pages at the end of the app summarising national neonatal guidelines		
4. Education	Educational text and images embedded throughout the app		

Prior to being implemented in Malawi and Zimbabwe central hospitals (19, 22), the NeoTree app was piloted in Zomba Central Hospital (ZCH), Malawi (20). This 1-month pilot included some preliminary usability testing which resulted in early adjustments to the app and produced the beta-app, “Minimum Viable Product 1” (MVP1), the most basic working version that can be safely launched and tested (23). Although mean SUS score for this version was 86.1 (24) (relatively high), this was in the context of a small preliminary phased pilot in a district general hospital (ZCH). Since usability varies with context (4) and NeoTree was now to be implemented permanently, in a new tertiary referral centre (Kamuzu Central Hospital (KCH), Malawi), the app now required further user-testing and refinement.

The overall aim of this study was therefore to investigate the usability of the NeoTree β-app (MVP1) from both qualitative and quantitative perspectives. Objectives were to first conduct qualitative (UXD/Agile) usability testing of MVP1 and apply themes to the usability-focused development of MVP2. Second, to conduct a quantitative usability testing of MVP1 using SUS. Third, to evaluate the usage of the NeoTree β-app (MVP2) during the first 6 months of deployment in a Malawian tertiary neonatal unit.

## Methods

### Setting

Malawi is a low-income country in sub-Saharan Africa with a high-neonatal mortality of 20 per thousand live births (25). This study was conducted in Ethel Mutharika Neonatal Unit at KCH, Lilongwe which is one of four Central hospitals in Malawi. Central hospitals in Malawi are tertiary referral centres providing the highest level of medical care. The neonatal unit admits 5–10 sick or small neonates each day with a capacity of 80-beds, serving a population of ~5 million people (26). The layout of the unit includes four separate wards: “High-risk” for unstable neonates requiring respiratory support, “Low-risk” for stable infants requiring feeding support, “Isolation” for patients requiring isolation, and a ward for Kangaroo Mother Care. There are 10 permanent neonatal staff providing care in the unit, such as continuous positive airways pressure, phototherapy, nasal cannula oxygen, and intravenous and oral medications. Surgery is available for common neonatal surgical problems including some congenital anomalies.

### Study-Design

**Figure 4:**
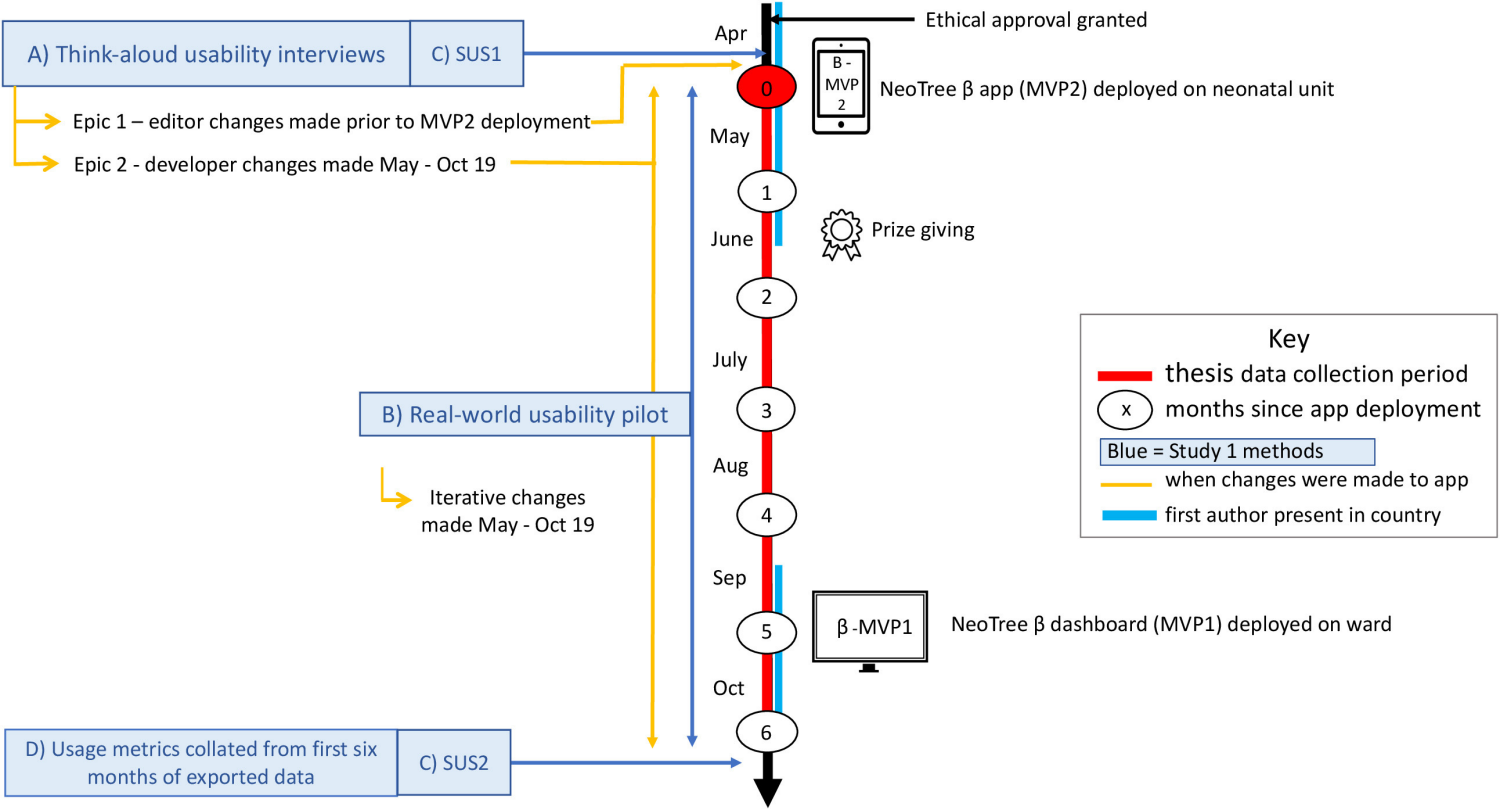
Study design.

This was a mixed methods study that included theoretical think-aloud (12) usability interviews prior to app deployment, a real-world usability pilot over 6 months, SUS scores collected before and after app deployment (baseline and end-line), and usage metrics exported from the app at the end of the pilot (Figure 4).

### Participants and Recruitment

Think-aloud interviews were conducted with 6 neonatal nursing staff prior to app deployment, because 85% of usability insights are revealed by user-testing with five participants (27, 28). Participants were recruited *via* WhatsApp using a purposive sampling method. Inclusion criteria were employment as a permanent member of neonatal nursing staff and no previous use of NeoTree app prior to the study. Exclusion criteria were any temporary staff or cadres not responsible for admitting neonates (e.g., clinicians). Information sheets were provided to potential participants in hardcopy or *via* WhatsApp. For the real-world pilot, participants were a convenience sample of any HCPs using the NeoTree during the first 6 months of rollout. For SUS we recruited 8 neonatal nurses [as sample sizes of 8 for SUS are 75% reliable (7)]. Inclusion criteria were that participants were permanent members of neonatal nursing staff, working at KCH. For baseline SUS1, they must have used the NeoTree app on just one occasion and for end-line SUS2 they must have used NeoTree during the real-world pilot. Exclusion criteria were any member of staff being paid by the NeoTree research team. For usage, participants were any HCP that used NeoTree during the real-world pilot.

### Procedure

**Figure 5:**
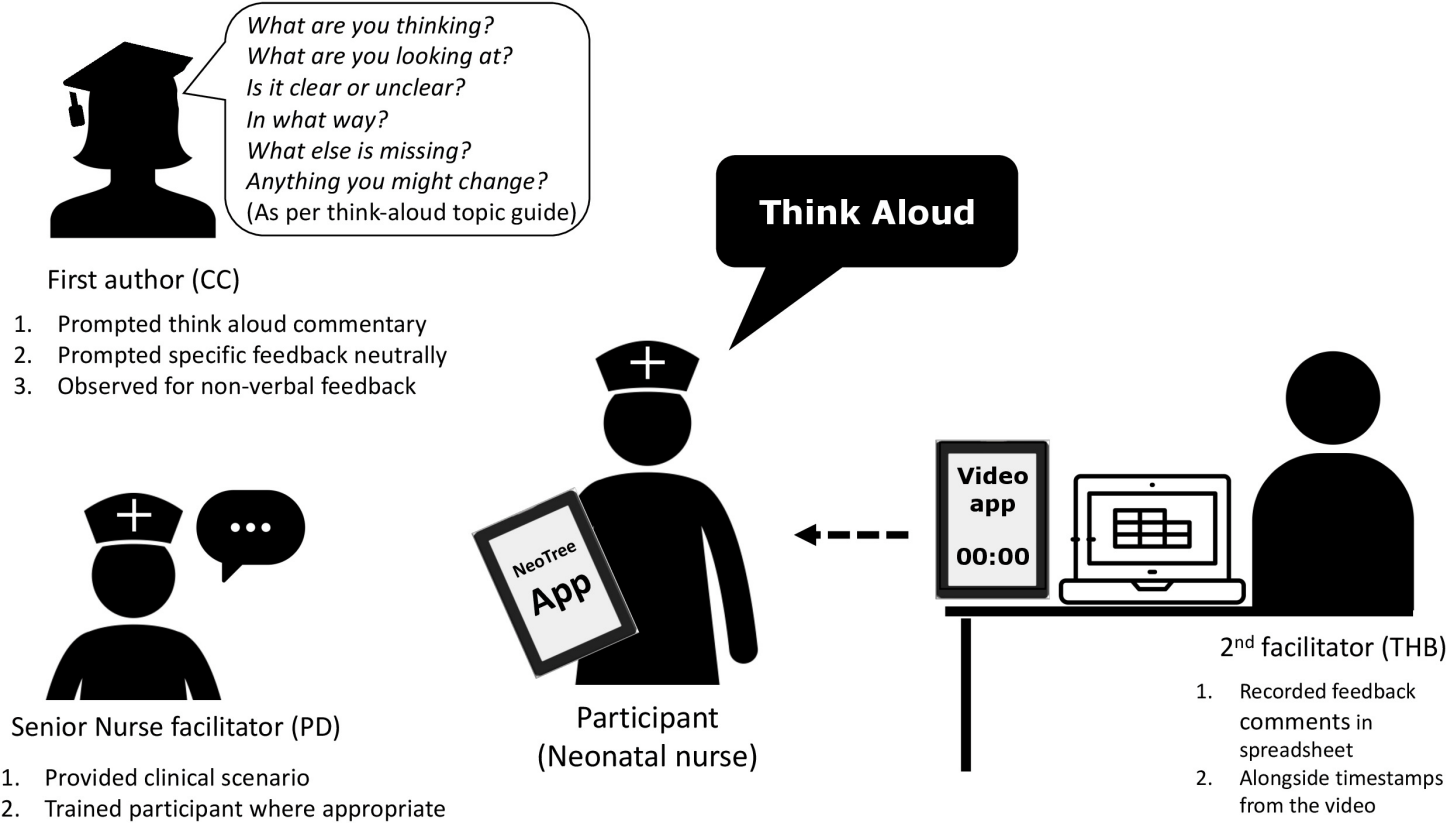
App usability interviews.

**Figure 6:**
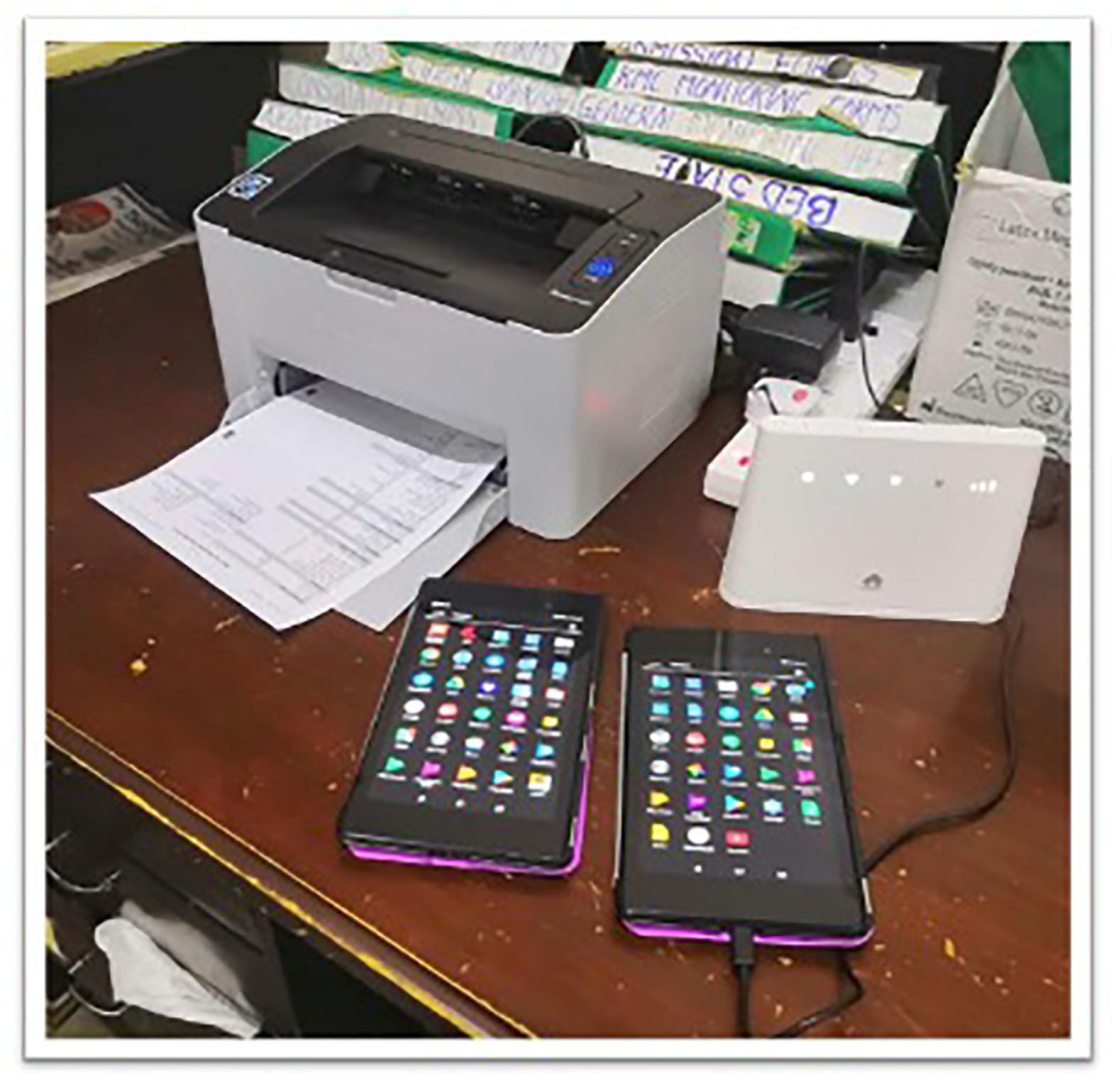
NeoTree hardware.

First think-aloud interviews were conducted with each participant as they “user-tested” the NeoTree β-app (MVP1) (Figure 5). Hardware are shown in Figure 6. Interviews were conducted by the first Author in English (the working language of Malawi) according to an adapted UX topic guide (Supplementary Material 1). After completing consent forms, each participant was assigned a participant number and asked to sit in the centre of the room, holding and looking at a tablet device containing the NeoTree app. Interviews were conducted in the absence of a sick neonatal patient; hence a senior neonatal nurse (PD) provided an imaginary patient scenario. Together, first author and neonatal nurse guided participants through a digital admission and discharge form, and how to print hard copies. This allowed participants to progress through the app, in a timely manner without struggling to think up example patient data. Whilst a degree of walking-through was required for Malawian participants, unused to theoretical testing, prompts were limited to non-leading questions, encouraging users to think-aloud and verbalise their thoughts as much as possible. A second researcher (THB) typed participants' voiced thoughts into an excel spreadsheet with timestamps, whilst video recording the session, making sure to capture both the participants hands and face as they used the app and the clock on the videoing device. Both the first author and second researcher could also voice observations or comments during the sessions, but they allowed participants to speak uninterrupted first, so as not to bias their responses. All three facilitators checked through the spreadsheet of usability notes for sense at the end of each session, adding any feedback that might have been missed. Upon leaving each usability interview, participants were issued with a training manual (to support their ongoing use of NeoTree), a certificate of attendance, and an appropriate cash remuneration (29).

Second, after making suggested changes from think-aloud sessions to the app, the real-world pilot was conducted. Six tablet devices containing the NeoTree app were deployed on the ward (at month 0: Figure 4) with chargers, extension leads, network router, and Wi-Fi printer. Users were informed that NeoTree would be a part of usual care and should be used for every baby at the point of admission and discharge, a policy supported by the heads of department (MC and YM). New users that had not attended the usability sessions were given induction training on the ward by a NeoTree ambassador and issued with a certificate and a training manual. User and patient data collected during the first 6 months of app deployment, were exported pseudo-anonymised from the tablets on a weekly basis to a cloud database, where admission and outcome forms were linked by NeoTree identification number. Three “NeoTree Ambassadors” already working in full time rolls (Nurse, Clinical Officer, and Paediatrician) at the hospital were paid a supplement to their usual salaries to support activities, provide technical support, supervision, and system maintenance. During the first 6 months of roll out, all users (permanent and temporary) were asked to report *ad-hoc* feedback regarding their experience using NeoTree either to the ambassadors or to the first author who recorded any feedback notes in a notebook and spreadsheet.

Thirdly, participants were asked to complete SUS score cards (4) (Supplementary Material 2), in the absence of the research team without signatures to maintain anonymity. Score cards included a Chichewa translation (the most frequently spoken Malawian language) as a helpful but not essential addition to ease understanding. These had been translated and back translated to check consistency.

Finally, four measures of usage were calculated (Table 2). For “user-count” and “cadre,” signatures and cadres recorded by HCPs at the end of NeoTree forms were exported. To provide a denominator for “coverage,” access to aggregate data from ward logbooks was granted by KCH's Health Management Information System department. For “completion-time,” users were asked to record in a ward notebook the start- and end-time of each NeoTree session they completed (displayed on session tabs in the app home page). These data were then copied from the logbook into an excel spreadsheet by an ambassador.

**Table 2:** Usage metrics.

**Measure**	**Definition**	**Calculated by**	**Programme**	**Assumptions / notes**
User count	The absolute number of individual health professionals who used NeoTree	Simple count (n)	Microsoft Power BI	
Cadre	The proportions of different cadres using NeoTree	Count of each cadre divided by total users (%)	Microsoft Power BI	
Coverage	The proportion of patient events recorded by the ward clerk on paper that were captured digitally on NeoTree	Total number of digital admissions divided by total number of admissions logged by ward-clerk (%) Total number of digital outcomes exported via NeoTree divided by total number of outcomes (%)	Microsoft Excel	Assumes there will be more patients logged on paper than admitted digitally. Compares totals only - does not match each individual name in the logbook with a digital admission or outcome
Completion-time	The time taken to complete a digital admission and outcome	Median number of minutes taken to complete admission (Median (IQR)) Median number of minutes taken to complete an outcome (Median (IQR))	Microsoft Excel	Data were exported for the whole 6 months Only last 2 months of data analysed (to allow for embedding of the intervention).

*Key: BI, Business Intelligence; IQR, Interquartile Range*.

### Analysis

**Figure 7:**
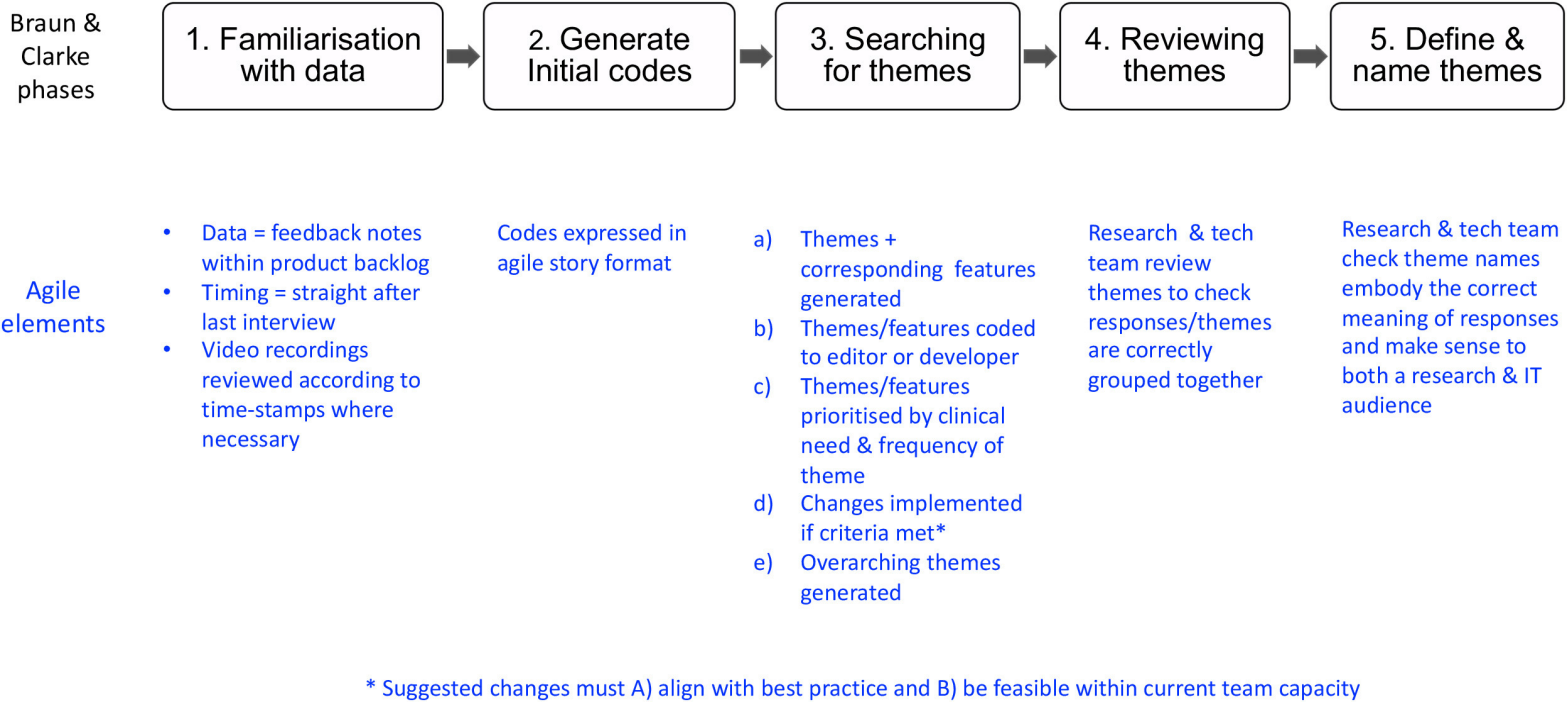
Rapid Agile analysis (Adapted from Braun and Clarke (30).

Analysis of the think aloud interviews combined Braun and Clarke's five thematic analysis phases (30) with the elements of Agile methodology (23, 31, 32) (Figure 7 adapted from Braun and Clarke). First, feedback notes (with timestamps) were transferred into the product backlog where the team could familiarise themselves with the whole dataset, re-consulting the videos where necessary. Second, the first author generated codes phrased as stories using the template “As a (Type of user) I want/need (some goal) so that (some reason) (32).” This approach not only captured why a piece of data was useful but also linked it to different user-roles (32, 33). For example, “As a neonatal nurse, I need Microcephaly in the head-shape dropdown list, so that I can accurately document head shape during an admission.” Third, similar codes were grouped together and assigned theme names which were then coded as “editor” (Epic 1) or “developer” (Epic 2) and prioritised within the backlog according to the clinical need/frequency of theme. App changes were then made in the order of the backlog, either configured by the first author (*via* the editor) or hard coded by the developers. Changes were only made if they (A) aligned with the best practice and (B) were feasible within team capacity. Overarching themes were then generated by grouping together similar themes under broader headings. In the fourth phase of analysis, themes and overarching themes were reviewed to check responses/themes that were correctly grouped together. Finally, theme names were defined with IT experts, to check that they embodied the correct meaning of the data and made sense to both a technical and research audience.

Usability feedback notes from the real-world pilot were analysed within the same backlog following the same 5-step process. Previous theme labels from the think-aloud interviews were used again where appropriate and corresponding changes implemented in the app iteratively as the pilot continued. Nurses were asked to briefly pause the use of app on the ward when changes were configured *via* the editor, to avoid crashes in the software. Median SUS [with interquartile range (IQR)] and mean SUS [with standard deviation (SD)] were calculated with 95% confidence intervals (*CI*s) for each time point. Median SUS scores were compared using Mann–Whitney *U*-test (if non-parametric) or mean SUS scores were compared using a *t*-test (if normally distributed). Finally, Microsoft Power-BI and Excel were used to analyse usage metrics as per Table 2.

## Results

Six participants attended usability interviews, eight completed SUS1, and eight completed SUS2 (participant details are included in Table 3). Those attending usability interviews included four women and two men, aged 28–43 years. Exact numbers of participants who gave *ad-hoc* feedback in the real-world pilot were not recorded.

**Table 3:** Demographic details and professional and training experience of participants attending think aloud app usability sessions (age and gender are excluded for confidentiality).

**Participant ID**	**A**	**B**	**C**	**D**	**E**	**F**	**G**	**H**	**I**	**J**	**K**	**L**
Cadre	NMT	RN	RN	RN	NMT	NMT	NMT	NMT	NO	NMT	RN	RN
Used tablet before?	N	Y	Y	Y	Y	Y	Y	Y	Y	Y	Y	Y
Uses tablet regularly?	N	Y	N	N	N	Y	Y	Y	Y	Y	Y	N
COIN/HBB training?	Y/N	Y/Y	Y/Y	Y/Y	Y/Y	Y/Y	Y/Y	Y/Y	Y/Y	N/N	N/N	Y/Y
Years of experience in newborn care	3	2	2	2	6	5	6	7	5	1	1	7
Attended think-aloud usability interviews	✓	✓	✓	✓	✓	✓	x	x	x	x	x	x
Completed SUS1	✓	✓	✓	✓	✓	✓	✓	✓	x	x	x	x
Completed SUS2	x	✓	x	✓	✓	✓	x	x	✓	✓	✓	✓

*NMT, Nurse Midwife Technician; RN, Registered Nurse; Y, Yes; N, No; COIN, Care of the infant newborn; HBB, Helping Babies Breathe; App, Application; SUS, System Usability Score*.

### Qualitative Usability

Overall, both think-aloud interviews and real-world pilot generated a total of 57 usability stories, 39 (68%) from think aloud analysis (Tables 4, 5), and 18 (32%) from the real-world testing (Table 6). In total, 21 usability themes/issues with corresponding app features were produced and features were added to the app (Tables 4–6). Themes were organised under 12 usability themes under two headings “Usability as an Electronic Medical Record (EMR)” and “Usability relating to clinical care.”

**Table 4:** Usability as an electronic medical record—Data collection feature set.

**Overarching usability theme**	**Usability themes**	**Corresponding features**	**#**	**Ed/Dev**	**A**	**B**	**Done**
	**With example feedback notes**	**And stories within each feature**					
**1. Exhaustiveness of data schema**	**Missing options in dropdowns/MCLs**	**Exhaustive field options**					
	*e.g. Microcephaly should be an option in head shape*	All relevant options included in dropdowns/MCLs	5	E	Y	Y	Y
	*e.g. Add flucloxacillin to medications list*						
	*If stillbirth selected include a prompt to fill in info as these babies not usually 'admitted' and we say 'Brought in Dead'*	Stillbirth or 'Brought In Dead' (BID) outcome option added	1	E	Y	Y	Y
	**Field type prevents complete data entry**	**Appropriate field type for complete data entry**					
	*Please allow multiple selections for reason for admission*	Reason for admission field changed to multiple choice	1	E	Y	Y	Y
	**Missing fields essential for an admission**	**Clinically exhaustive set of fields**					
	*Chlorhexidine for umbilicus is needed*	Chlorhexidine field added	2	E	Y	Y	Y
**2. Prevention of errors to support data integrity**	**Lack of field validation:**	**Field validation**					
	*Dashes are hard to find on the keyboard for NeoTree ID—suggest take out dash and stop us entering wrong characters*	Field validation added to ID number field on discharge so dash already present & wrong characters cannot be entered	3	D	Y	Y	Y
	*Date field on discharge form needs to allow dates in the future*	Field validation *via* editor to allow only dates in the future/past	1	D	Y	N	N
	**Contradictory field options:**	**Exclusive field options**					
	Symptom review—crying > normal & Crying < normal needs to be exclusive	Exclusivity option *via* editor to prevent selection of 2 contradictory MCL options	1	D	Y	N	N
**3. Ease of progression through the app**	**Compulsory fields difficult**	**Non-compulsory field to allow progression**					
	*Apgar's shouldn't be compulsory as for referrals they are often not available*	Apgar field made non-compulsory so HCPs can continue even when apgars are not available	3	E	Y	Y	Y
	**Absence of 'Unknown' field option**	**Unknown option to allow progression**					
	*Birth History—“Unknown” option for TEO and vit K*	Included when necessary so HCPs can progress even when information not available	2	E	Y	Y	Y
**4. Efficiency of data entry using shortcuts/calculations**	**Inefficient data entry due to sub-optimal field type**	**Field-types to create shortcuts**					
	*Drop down of permanent staff for signature on discharge form*	Signature field changed to a dropdown at end of both admission & discharge forms making sign-off quicker	1	E	Y	Y	Y
	*Follow up date entry on discharge is laborious*	Future date field-type to allow selection of future dates from a calendar (so HCPs can schedule follow up clinics	4	D	Y	N	N
	**Lack of simple calculations**	**Advanced field logic:**					
	*Age calculation for all babies?*	Calculation of age for babies at all ages, including >7 days	1	D	Y	N	N
**5. Navigation of user interface**	**Lack of instructions**	**Calls to action:**					
	*Respiratory support page—can you click multiple options?*	Reminder '*Click all that apply*' added to all multiple-choice lists so HCPs know they can enter more than 1 option	5	E	Y	Y	Y
	*Feeding page—the multiple selection is not obvious*						
	*Apgar field needs “If known”*	Caveat messages added where necessary e.g., “*if available*” or “*if present*”, or “*only blue fields need completing*” to pages where fields are non-compulsory	2	E	Y	Y	Y
	*In modifiable factors—can you put “If present please fill in”*						
	*Tap to start on clock not immediately obvious*	Instructions “TAP TO START” made larger in size	1	D	Y	Y	Y
	Tries to tap the page with tasks—consider “when completed tasks continue”	Add instruction to “tasks” and “navigation” pages that HCPs only need to click the continue button	1	E	Y	Y	Y
	*Struggled with scrolling*	Instructions explaining how to scroll	2	E	Y	N	N
	*Hesitation on scrolling up*						
	Vital signs—number keypad obscures input	Instructions on how to close number keypad	1	E	Y	N	N
	**Lack of signposting**	**Signposting**					
	*Maternal history—Churches signpost it's in alphabetical order*	Signpost added to list of churches is in alphabetical order	1	E	Y	Y	Y
	*Tell user secondary diagnoses can be added later in form on primary discharge page*	Add “secondary diagnoses can be added later” on primary diagnosis page	1	E	Y	Y	Y
	*Put HW ID instruction in field not page title*.	Put example HW-ID in field title rather than on page but doesn't fit in field title, and clearly explained in page content	1	E	Y	N	N
	**Confusing layout/design**	**UI design**					
	*tried to click writing instead of text box*	Make answer boxes immediately obvious/highlighted	2	D	Y	Y	Y
	*Confused as lines are grey even for blue fields? Can lines be blue?*	Make lines for active fields blue consistent with the colour of the writing of active fields	1	D	Y	Y	Y
	*Confusion between grey/blue inputs—have a star or something to show next compulsory input*	Include some indication on UI which fields are compulsory and which fields are non-compulsory.	3	D	Y	Y	Y
	*kept trying to click navigation bar (on a non-click page)*	Distinguish 'click' pages from 'non-click' pages with different colours	5	D	Y	N	N
	*Pages without input fields i.e., navigation pages—different colour?*						
	*Delete button confused with done button—suggest increase size of done button?*	Bigger/brighter continue button	1	D	Y	N	N
**6. Relevancy of content**	**Unnecessary field options:**	**Remove irrelevant field-options**					
	*We don't have Intra-nasal/head box oxygen at KCH*	Remove intra-nasal & headbox oxygen options from respiratory support field on discharge	1	E	N	Y	N

*Red font, Outstanding developer adjustments as of Nov 2019; Blue font, outstanding editor adjustments as of Nov 2019; #, number of mentions by participants; Ed, Editor; Dev, Developer; A, criteria A: Aligns with clinical best practice; B, criteria B: practical and feasible to implement within team's capacity*.

**Table 5:** Usability relating to clinical care—clinical care feature set.

**Overarching usability theme**	**Usability themes**	**Corresponding features**	**#**	**Ed/Dev**	**A**	**B**	**Done**
	**With example feedback notes**	**And stories within each feature**					
**7. Confidentiality of identifiable information**	**Lack of confidentiality**	**Confidential fields:**					
	*Babies name should be confidential on the discharge*	All patient identifiable fields in the discharge form made confidential so they are not exported to the database	1	E	Y	Y	Y
**8. Cohesion with usual ward process**	**Lack of elements of usual admission process**	**New pages to match ward process:**					
	*Can you print management plan?? And include in main app*	Overall admission management plan page added to admission form	2	E	Y	Y	Y
	*Abnormal looking umbilicus—(does that include exomphalos/gastroschisis??)*	Exomphalos and gastroschisis management page added	1	E	Y	Y	Y
	*Health education page at the end of the discharge?*	Health promotion for mothers/guardians page added to end of discharge	1	E	Y	Y	Y
**9. Embedded educational content**	**Lack of required educational text**	**Educational text:**					
	*Can't remember early & late-stage cut-off for sepsis*	Clear explanation/reminder of what is early & late neonatal sepsis added to diagnosis at discharge page	1	E	Y	Y	Y
	**Lack of required educational images**	**Educational images:**					
	*Method of checking the tone should be reinforced*	Pictures added re how to measure tone on admission	1	E	Y	Y	Y
**10. Locally coherent language**	**Locally inappropriate language**	**Locally understandable language**					
	*Birth History—“Vit K given at birth”*	e.g., Specify vit K “given at birth”	5	E	Y	Y	Y
	*Light palpation of abdomen rather than softly*	e.g., Change from “softly” to “lightly” palpate the abdomen					
	* < or > not understood on 18 hrs*	e.g., Remove > or < symbols—write out					
	*Still birth—leave in form*	e.g., Discuss nomenclature for still births & BIDs with team					
	*Adjust maturity score to say Ballard??*	Change 'maturity score' to ‘Ballard score'	3	E	N	Y	N
	*No one is using the coin maturity score*						
	*FeFo write out—as iron and folate—some HCWs who are not midwives by training don't know this*	Write out 'FeFo' (Ferrous sulphate &Folate)	3	E	Y	Y	Y
**11. Adaptability of UI according to resources**	**Lack of resources/confidence using resources required to complete app**	**Configuration page**					
	*Users probably happy with stet for lungs but not heart*	Add configuration options to editor so the app can be tailored to availability of resources e.g., Stethoscopes, Tape measures, by the nurse in charge.	3	E/D	Y	Y	Y
	*Not confident with Stethoscope—needs training*						
	*Tape measures may not be available*						
**12. Print-out design to facilitate handover**	**Printout heading confusing**	**Clear printout headings**					
	*Double heading of diagnosis on admission print out*.	adjusted on print out to facilitate easy hand over process e.g., remove extra diagnosis section heading	1	E	Y	Y	Y
	**Difficult to see abnormal data on printout**	**Data highlighting on printout:**					
	*highlight abnormalities on the printout—editor needs bold/colour capability*	Highlight “abnormal” data/important fields on the print-out to facilitate easy handover	3	D	Y	N	N
	*Highlight BIRTH DATE not admission date on printout*						
	*Highlight data on admission that needs to be entered into Discharge form*						

*Red font, Outstanding developer adjustments as of Nov 2019; Blue font, outstanding editor adjustments as of Nov 2019; #, number of mentions by participants; Ed, Editor; Dev, Developer; A, criteria A: Aligns with clinical best practice; B, criteria B: practical and feasible to implement within team's capacity*.

**Table 6:** Usability of the NeoTree beta app – iterative changes made during real-world pilot.

**Usability themes findings—generated iteratively during first 6 months of rollout**							
**Overarching usability theme**	**Usability Themes**	**Corresponding features**	**#**	**Ed/Dev**	**A**	**B**	**Done?**
	**With example feedback notes**	**And stories within each feature**					
**Usability— data collection feature set**							
**1. Exhaustiveness of data schema**	**Missing options in dropdowns/MCLs**	**Exhaustive field-options**					
	*Please include option for 4xSP doses in antenatal secti*	All relevant option included in MCLs & Dropdowns	1	E	Y	Y	Y
	*Please include option for Respiratory distress of the newborn (term) in diagnosis list*		1	E	Y	Y	Y
	*Unrecordable option for blood sugar??*		1	D	Y	N	N
	*Cardiac clinic on Thursdays as follow up option?*		1	E	Y	Y	Y
	*Congenital abnormalities should include Hydrocephalus, Spinal deformity, cleft lip & palate, ano-rectal malformation*		1	E	Y	Y	Y
	*Outcomes on the discharge should include discharged, Absconded, transferred to other ward, transferred to other hospital, NND <24 hrs and NND > 24 hrs*		1	E	Y	Y	Y
	**Missing fields essential for newborn admission**	**Clinically exhaustive set of fields:**					
	*Include examination of the palate in examination section*	Palate field added so HCPs can document examination of palate	1	E	Y	Y	Y
	*Please include no. of sibling's dead field as this was on old MOH form*	Number of sibling's dead field not included (as removed previously)	1	E	N	N	N
	*BID should have a focused Hx and exa*	New fields added to BID/Stillbirth script	1	E	Y	Y	Y
	*Can you include a cause of BID/Stillbirth field?*		1	E	Y	Y	Y
	*Can you include modifiable factors for babies BID/Stillbirth?*		1	E	Y	Y	Y
**3. Ease of progression through app**	**Compulsory fields sometimes difficult**	**Non-compulsory fields:**					
	*Can you grey out the patient 1st name and surname for BID/Stillbirths?*	Make name fields optional or greyed out completely for BIDs	1	E	Y	Y	Y
**5. Navigation of UI**	**Lack of instructions**	**Calls to action:**					
	*How do we enter the name of dumped baby when they have no name?*	Instructions included on how to name a dumped baby	1	E	Y	Y	Y
**Usability—clinical care feature set**							
**9. Embedded education & decision support**	**Lack of educational text**	**Educational text:**					
	*Good to show basic comparison table on surgical Management page*	Table added to surgical gastroschisis/exomphalos page indicating how to distinguish between the two diagnoses	1	E	Y	Y	Y
	*Include management of gastroschisis vs. exomphalos*	Management page for gastroschisis/exomphalos revised after surgical review	1	E	Y	Y	Y
	**Lack of educational images**	**Educational images:**					
	*We need a picture of 'strong distal flexion' in Thompson score*	Picture of strong distal flexion	1	E	Y	N	N
	*Picture of weighing baby naked would remind us to weigh baby naked!*	Picture of weighing baby naked	1	E	Y	N	N
	*Picture of how to do measure OFC properly would be helpful*	Picture of measuring OFC	1	E	Y	N	N

*Red font, Outstanding developer adjustments as of Nov 2019; Blue font, outstanding editor adjustments as of Nov 2019; #, number of mentions by participants; Ed, Editor; Dev, Developer; A, criteria A: Aligns with clinical best practice; B, criteria B: practical and feasible to implement within team's capacity; MCL, Multiple Choice List; NND, Neonatal death; BID, Brought in dead; OFC, Occipito-Frontal Circumference; MOH, Ministry of Health*.

Six overarching themes related to usability as an EMR (i.e., the data collection function of NeoTree) were generated (Table 4—themes 1–6) with the most common being “exhaustiveness of data schema.” Example of adjustments included changing the field type, e.g., the “reason for admission” field was changed from single choice to multiple-choice because sick newborns often present with more than one problem. Other additions included important missing fields (e.g., a question regarding whether chlorhexidine had been applied to the umbilicus after birth) and relevant missing options in dropdown lists. Other key changes to the EMR included improved field validations, e.g., restricting the patient identification number to certain digits (prevention of errors to support data integrity), making non-essential fields optional or adding “unknown” options (ease of progression through app), advanced field logic to automatically calculate the age of the baby (efficiency of data entry using shortcuts/calculations), removing irrelevant confusing form content (relevancy of content), and adding instructions and signposts to improve navigation (navigation of user interface [UI]). Real-world testing revealed problems with entering babies brought in dead and prompted making the name field optional for these cases as usually they are not given a name.

Six overarching themes relating to the clinical usability of NeoTree were generated, such as confidentiality of identifiable information, cohesion with usual ward processes, embedded education, locally coherent clinical language, adaptability of UI according to available resources, and printout design to facilitate handover (Table 5—themes 7–12). Key examples of changes made to improve the clinical usability of NeoTree included adding a page with common management options and a specific page to facilitate the pathway for surgical babies with gastroschisis/exomphalos (cohesion with usual ward process). Other examples of clinical usability improvements included adding an explanation of the time cut-off between early and late neonatal sepsis and a picture of “strong distal flexion” from the Thompson score for neonatal encephalopathy (embedded education) and the ability for nurses to turn-off the head circumference question when tape-measures were not available (adaptability of UI to available resources). Again, real-world testing resulted in more nuanced iterative refinements, e.g., the addition of the surgeons' contact details to the new gastroschisis page.

### SUS

Mean SUS1 prior to the app adjustments was high at 88.1 and SUS2 rose marginally to 89.4 (Table 7). Both scores remained in the 80–90 range with no statistically significant difference between sample means (*p* = 0.389).

**Table 7:** SUS responses and scores.

**Responses to SUS**		
**Questions are answered using a likert scale of 1–5 where 1 = strongly disagree and 5 = strongly agree**	**SUS1** **(After 1 use of NeoTree App, pre app usability adjustments)**	**SUS2** **(After 6 months of NeoTree use, post app usability adjustments)**
**Question**	**Mean (SD)**	**Mean (SD)**
1. I think I would like to use this system frequently	4.8 (0.5)	4.8 (0.5)
2. I found the system unnecessarily complex	1.5 (1.1)	1.3 (0.5)
3. I thought the system was easy to use	4.8 (0.5)	4.8 (0.5)
4. I think that I would need the support of a technical person to be able to use this system	1.3 (0.5)	1.4 (0.7)
5. I found the various functions in this system were well-integrated	4.6 (0.5)	4.6 (0.7)
6. I thought there was too much inconsistency in this system	2.0 (0.5)	1.3 (0.5)
7. I would imagine that most people would learn to use this system	4.4 (0.5)	4.1 (1.5)
8. I found the system very cumbersome to use	1.9 (0.5)	1.9 (1.0)
9. I felt very confident using the system	5.0 (0.5)	4.9 (0.4)
10. I needed to learn a lot of things before I could get going with this system	1.6 (0.5)	1.6 (0.7)
Overall SUS score^*^	88.1 (10.2)	89.4 (7.0)

*SUS, System Usability Score; IQR, Interquartile range; SD, Standard Deviation*.

* *Overall mean SUS score calculated by subtracting 1 from odd numbered question scores, subtracting even numbered question scores from 5 and then adding them up and dividing the total by 10*.

### User-Count

Ninety-three different individuals used NeoTree ß-app during the first 6 months of implementation including 9 permanent staff and multiple temporary staff and students. The 9 permanent staff working on the unit had used the NeoTree app on average 96 times (range 51–195) by the end of the first 6 months.

### User-Cadre

Out of 1,323 admissions, cadre data were available for 1,181 admissions which were completed by five different cadres: Nurse Midwife technicians (417, 35%), Nursing Officers (498, 42%), Nursing Students (260, 22%), Medical Students (1, 0.08%), and Clinical Officers (5, 0.42%). Hence, almost all admissions were completed by nursing cadres, one-fifth by students.

### Coverage

The NeoTree app captured 1,323 digital admissions compared with 1,298 paper admissions logged by the ward-clerk (25 more admissions *via* the app = 102% of paper admissions). The NeoTree captured 1,197 digital outcomes compared with 1,180 paper outcomes logged by the ward-clerk (17 more outcomes *via* the app = 101% of paper outcomes). If “coverage” is the proportion of paper-recorded patient-events captured digitally by NeoTree, then coverage was 100% for both admissions and outcomes with NeoTree events slightly exceeding those logged on paper. Matched outcomes were achieved for 1,197 (90% of NeoTree admissions) leaving 10% with no digital outcome in the database.

### Completion Time

The median completion time for NeoTree admission and outcome forms during the last 2 months of the study was 16 (IQR 11.21) and 8 (IQR 5.12) minutes respectively.

## Discussion

In this study, we have demonstrated usability-focused optimisation of an app for neonatal HCPs in theoretical and real-world conditions. Rapid Agile analysis allowed abundant, detailed, context-specific, user-led refinements to be applied rapidly and iteratively, expediting deployment, and optimising usability. A new MVP2 was produced, and this was used widely on a low-resource neonatal ward, achieving high usability scores and coverage with short completion times. Multiple usability priorities relating to NeoTree as an EMR, and a clinical aid were uncovered and acted on. Similar bedside apps for low-resource settings may also benefit from this approach to prevent later implementation problems.

Results of this 2019 study can be directly compared with the previous smaller 1-month pilot in 2017 (Table 8). This larger 6-month study included more users (93 vs. 46), achieved higher SUS (89.4 vs. 86.1), higher coverage (100 vs. 70%), and shorter completion times (16 vs. 37 min for an admission). Together these might suggest that the MVP2 produced in this study was more usable than the previous MVP1. However, it is difficult to know the contribution of other factors in this new facility setting, such as the policy to use NeoTree on every baby, and the employment of NeoTree champions to provide technical support. Some of the usability themes and features from the pilot were similar to those of this study, however, this analysis was more sophisticated organising and sorting themes more clearly. Occasionally the same features were added in this study as were previously added (e.g., adding unknown options and making questions non-compulsory to ease progression through the app), but this time they were added to all parts of a now more complex app. Optimising usability in this way without compromising data integrity was more of a challenge here, as we approached the minimum dataset required for each baby. Complete data were now also crucial for quality improvement *via* new features of the system (diagnostic algorithm and connected dashboard). This trade-off between usability and data integrity could be a relevant consideration for other apps that combine data capture with quality improvement.

**Table 8:** Comparison of findings with previous pilot.

			**Previous pilot (2017)**	**This study (2019)**
**Aim**	To develop NeoTree from x –> y		Alpha prototype –> MVP1	MVP1 –> MVP2
**Method**	**Setting**		Zomba Central Hospital (ZCH) District level hospital—Southern Region of Malawi, permanent neonatal staff = 20	Kamuzu Central Hospital (KCH)—Lilongwe Tertiary referral centre—Central Region of Malawi, permanent neonatal staff = 10
	**Procedure:**	Think aloud interviews *(n)*	13	6
		Real-world pilot	1 month of use phased in over time	6 months using NeoTree as part of usual care
			NeoTree completed in addition to paper, on a temporary basis—for duration of study only	NeoTree completely replaced paper, on a permanent basis policy to use NeoTree on all neonates
			No technical support in place Author on site 9–5 Mon to Fri	3 × NeoTree Ambassadors present Author on site for 1st & last month only
**Results**	Total participants *(n)*		43	93
	Qualitative usability themes (*n*)		11	12
	Qualitative usability theme names		1. *Type of question* 2. *Sequence of fields* 3. *Language* 4. *Completing fields* 5. *Using timer* 6. *Understanding instructions* 7. *Length of question* 8. *Information not available* 9. *Proceeding through the app* 10. *Navigation* 11. *Drop-down menus*	Themes relating to data capture: 1. *Exhaustiveness of data schema* 2. *Prevention of errors to support data integrity* 3. *Ease of progression through the app* 4. *Efficiency of data entry using shortcuts/calculations* 5. *Navigation of user interface* 6. *Relevancy of content* Themes relating to clinical care: 7. *Confidentiality of identifiable information* 8. *Cohesion with usual ward process* 9. *Embedded educational content* 10. *Locally coherent clinical language* 11. *Adaptability of UI according to resources* 12. *Print-out design to facilitate handover*
	SUS		80.8 –> 86.1 (*n* = 13.13)	88.1 –> 89.4 (*n* = 8.8)
	NeoTree admissions captured *(n)*		134	1,323
	NeoTree outcomes captured *(n)*		129	1,197
	Coverage of actual admissions (%)		70	100
	Completion time—admissions (min)		Mean = 37 (range 18–59)	Median = 16 (IQR 11, 21)
	Completion time—outcomes (min)		n/a—(completed by the authors)	Median = 8 (IQR 5, 12)
	User cadre		Mainly nursing cadres (53% students)	Mainly nursing cadres (22% students)

Expected usability themes produced in this study relating to NeoTree's data capture function, included “Exhaustiveness of data schema.” An exhaustive digital form that includes all relevant data fields and all possible list options is likely to reduce user frustration and forced errors, while providing a structured, standardised, and systematic user experience. Similar themes from other app studies include that the mPneumonia app allowed users to systematically capture all signs and symptoms of pneumonia (34), a Dutch digital intake tool produced a better overall picture of patients (35) and an Australian EMR prevented nurses from missing out important data inputs (36). The “relevancy of content” theme was also expected because this is consistent with lean product development which recommends that efficient systems should minimise waste (37). This in turn aligns with data protection requirements that data systems should only collect the minimum patient data needed to successfully accomplish a given task (38). For these reasons and to minimise possible harm from the burden of data collection (39), our study supports that adding new fields to clinical apps for low resource settings, should be kept to a minimum and recommends the creation of national minimum datasets already underway in Malawi.

Expected “clinical usability' improvements included adding extra pages to digitally replicate the original paper forms” (theme “cohesion with usual ward process”) aiming to reduce deviation from norms and increase buy-in. This theme corroborates a review of EMR-powered clinical decision support systems, which concluded that integrating new technologies into clinical work practices is essential for successful implementation (40). Moreover, adding educational images to improve clinical usability, was helpful for an app for illiterate midwives in Guatemala, substantiating that picture content may improve usability in low resource settings where there may be reduced literacy, or multiple languages spoken. In addition, “locally coherent clinical language” was true for apps in high resource settings where usability themes included “clarity of wording” (41) and “clear and unambiguous text feedback” (42), highlighting the importance of clear, context-specific written communication for health apps across settings.

Although SUS scores for NeoTree ß were expected to be high (following relatively high scores in the pilot), the lack of statistically significant rise in SUS after adjustments might be explained by four of the same staff completing both SUS1 and SUS2, or a possible inflation of baseline scores and therefore less room for improvement. High baseline SUS scores could have been due to a more competent group of staff using the app this time, or initial excitement and enthusiasm for a new intervention. Indeed, the perceived newness and modernity of the mPneumonia app in Ghana improved acceptance and was associated with the perception of improved care (34). Overall, NeoTree's SUS scores have been consistently higher than those of UK Emergency Department (ED) data systems, which recently scored below average (median SUS 53) (3) suggesting NeoTree may be easier to use than these. However, the UK study compared a much larger array of digital systems, while in Malawi NeoTree is the first such system, hence with no comparisons available subjective SUS scores might be inflated.

While think aloud feedback and SUS scores are both the subjective measures of usability, our objective usage statistics were encouragingly high after refinements were made, adding weight to the qualitative aspects of the study, and demonstrating that NeoTree MVP2 was used widely. The proficiency or efficiency of use, may be indicated by shorter completion times compared with the Zomba pilot (20), but these may also be attributable to more practice time over a 6-month period. In this study similar to the pilot, a significant proportion of admissions (one-fifth) were completed by nursing students which may reflect the apps educational advantages (20) or an ongoing reliance on students for service provision, in a resource-limited setting. The problem of staff shortages endures across African healthcare settings (43–46), and needs more consideration by Governments and NGOs as new technologies are introduced.

## Limitations

A limitation of the study might be the reliance on the subjective verbal expressions of a small number of users to guide and shape the application as per Agile. Objective log data of user errors (such as, completing a field and then deleting it or navigating to a page and then navigating back) might strengthen the study. However, log data analytics were not feasible within team capacity in a preliminary phase of the project. Despite the small sample size, qualitative data were rich, prompting abundant app adjustments. Log data analytics could potentially be made available in the future to strengthen follow on usability studies, with perhaps a more structured task analysis, but these fail to capture human idiosyncrasies and require large samples for statistical relevance. Agile methods allow lean iterative development at accelerated velocity, focusing on usability and outcomes with only a few participants, and are mainstream in industrial organisations. Increasingly academic publications are employing Agile methods. For example, a Kenyan mixed-method study combined qualitative interviews with human centred design workshops to engage users in designing and tailoring an intervention to fit the low-resource context (47).

Other limitations include that the interviews were not transcribed verbatim, and this might have limited the qualitative analysis by allowing bias during summarisation or interpretation of participant responses. However, the interview dialogue was relatively sparse and usability notes often related to observed interactions with the UI (such as clicking the wrong button), hence it was decided that transcribing the videos would not add significantly to the richness of data. Completion times were recorded manually from the app home page that may have been affected by human error. These data are amenable to automation in future studies.

## Conclusion

This study show-cases usability-focused development of a digital app for neonatal HCPs in a low-resource neonatal unit, combining Agile methods with thematic analysis to expedite deployment and allow iterative user centric refinement during real-world use. System usability remained high after refinements were made and usage was high with 93 neonatal nurses using the app on 1,323 babies. Coverage of admissions and discharges exceeded numbers recorded on paper and completion times were shorter than a previous pilots. Results suggest that NeoTree was now tailored enough for successful use on a permanent basis and the large number of qualitative usability insights and subsequent refinements, promotes the importance of usability for digital intervention development alongside acceptability and feasibility. This study could inform the optimisation, uptake, and implementation of similar apps for other low-resource hospitals as they digitalise over the coming decades.

## Data Availability Statement

The data analysed in this study are part of an MDRes dissertation at UCL. For more information please contact the corresponding author.

## Ethics Statement

Ethical approval for this study was confirmed with the University of Malawi, College of Medicine Research Council (P.02/19/2613) and the UCL Research Ethics Board (Ref. 6681/001).

## Author Contributions

CC conceptualised and conducted the study, analysed the data, and wrote the manuscript. MC is Malawi principle investigator, advised on study design, assisted with ethical approval, supported app implementation, and revised the manuscript. YM was chief NeoTree Ambassador overseeing KCH NeoTree activities and edited the manuscript. PD was also NeoTree Ambassador, facilitated usability interviews, contributed to analysis, and supervised the real-world pilot. TH-B was project manager, facilitated usability interviews, contributed to analysis, and oversaw NeoTree logistics. CN contributed to the conceptualisation and study design, analysis and edited the manuscript. YS advised on data analysis. DN contributed to data cleaning and quantitative analysis. KG contributed to analysis by reviewing usability themes. ML, FL, and MH supervised CC in her MD thesis, advising on study design and methodology, supervising CC in the field (remotely), and editing the manuscript. MH is principal investigator and contributed to the conceptualisation of the study. All authors contributed to the article and approved the submitted version.

## Funding

This study was completed as part of an MDRes thesis for CC which was funded partly by a clinical fellow post at UCL, the Naughton Cliffe Matthews grant (PI MH) and partly by charity donations to the NeoTree charity. This research was funded in part by the Wellcome Trust [215742_Z_19_Z]. For the purpose of open access, author has applied a CC BY public copyright license to any Author Accepted manuscript version arising from this submission.

## Conflict of Interest

CN was employed by Spinspire Consultants. KG acquired her Agile certification from ICAgile but was not employed by ICAgile at the time of this work. YS was employed by Snowplow Analytics. The remaining authors declare that the research was conducted in the absence of any commercial or financial relationships that could be construed as a potential conflict of interest.

## Publisher's Note

All claims expressed in this article are solely those of the authors and do not necessarily represent those of their affiliated organizations, or those of the publisher, the editors and the reviewers. Any product that may be evaluated in this article, or claim that may be made by its manufacturer, is not guaranteed or endorsed by the publisher.

## Abbreviations

App: Application
ED: Emergency Department
HCH: Harare Central Hospital
HCP: Health Care Professional
KCH: Kamuzu Central Hospital
MCL: Multiple Choice List
MVP: Minimum Viable Product
SUS: System Usability Score
UCD: User Centred Design
UX: User Experience
UI: User Interface
ZCH: Zomba Central Hospital.

## References

[1] Home, | Usability,.gov. Available online at: https://www.usability.gov/ (accessed January 9, 2020).

[2] Jaspers MWM. A comparison of usability methods for testing interactive health technologies: methodological aspects and empirical evidence. Int J Med Inform. (2009) 78:340–53. 10.1016/j.ijmedinf.2008.10.002 19046928

[3] Bloom BM Pott J Thomas S Gaunt DR Hughes TC. Usability of electronic health record systems in UK EDs. Emerg Med J. (2021) 38:410–5. 10.1136/emermed-2020-210401 33658268 PMC8165140

[4] Brooke J. SUS - A quick and dirty usability scale. Usability Eval Ind. (1996) 189:4–7.

[5] Bangor A Kortum PT Miller JT. An empirical evaluation of the system usability scale. Intl J Hum Computer Interact. (2008) 24:574–94. 10.1080/10447310802205776

[6] Kortum PT Bangor A. Usability ratings for everyday products measured with the system usability scale. Int J Hum Comput Interact. (2013) 29:67–76. 10.1080/10447318.2012.681221

[7] Tullis TS Stetson JN. A Comparison of Questionnaires for Assessing Website Usability ABSTRACT: Introduction. Usability Prof Assoc Conf (2004) 1–12.

[8] Martins AI Rosa AF Queirós A Silva A Rocha NP. European Portuguese validation of the System Usability Scale (SUS). In: Procedia Computer Science. Sankt Augustin: Elsevier B.V. (2015). p. 293–300. 10.1016/j.procs.2015.09.273

[9] Peres SC Pham T Phillips R. Validation of the system usability scale (sus): sus in the wild. In: Proceedings of the Human Factors and Ergonomics Society. Los Angeles, CA: SAGE PublicationsSage CA (2013). p. 192–6.

[10] Sauro J. Measuring Usability With The System Usability Scale (SUS). Userfocus (2016). Available online at: https://www.userfocus.co.uk/articles/measuring-usability-with-the-SUS.html (accessed May 4, 2021).

[11] Rohrer C. When to Use Which User-Experience Research Methods. Nielsen Norman Group (2014). p. 1–7. Available online at: http://www.nngroup.com/articles/which-ux-research-methods/ (accessed October 05, 2019).

[12] Broekhuis M van Velsen L Hermens H. Assessing usability of eHealth technology: a comparison of usability benchmarking instruments. Int J Med Inform. (2019) 128:24–31. 10.1016/j.ijmedinf.2019.05.001 31160008

[13] Agile and User-Centered Design | ThoughtWorks. Available online at: https://www.thoughtworks.com/insights/blog/agile-and-user-centered-design-0 (accessed April 20, 2021).

[14] Modave F Bian J Rosenberg E Mendoza T Liang Z Bhosale R. DiaFit: the development of a smart App for patients with type 2 diabetes and obesity. JMIR Diabetes. (2016) 1:e5. 10.2196/diabetes.6662 29388609 PMC5788459

[15] Sandhu H Wilson K Reed N Mihailidis A. A mobile phone app for the self-management of pediatric concussion: development and usability testing. J Med Internet Res. (2019) 21:e12135. 10.2196/12135 31152527 PMC6658289

[16] Rivera J McPherson AC Hamilton J Birken C Coons M Peters M. User-centered design of a mobile app for weight and health management in adolescents with complex health needs: qualitative study. J Med Internet Res. (2018) 2:e7. 10.2196/formative.8248 30684409 PMC6334679

[17] Vilardaga R Rizo J Zeng E Kientz JA Ries R Otis C. User-centered design of learn to quit, a smoking cessation smartphone app for people with serious mental illness. J Med Internet Res. (2018) 6:e2. 10.2196/games.8881 29339346 PMC5790963

[18] Martinez B Hall-Clifford R Coyote E Stroux L Valderrama CE Aaron C. Agile development of a smartphone app for perinatal monitoring in a resource-constrained setting. J Health Inform Dev Ctries. (2017) 11:1–2. Available online at:http://www.jhidc.org/index.php/jhidc/article/view/158/212 28936111 PMC5604479

[19] Mgusha Y Nkhoma DB Chiume M Gundo B Gundo R Shair F. Admissions to a low-resource neonatal unit in malawi using a mobile app and dashboard: a 1-year digital perinatal outcome audit. Front Digit Heal. (2021) 3:761128. 10.3389/fdgth.2021.761128 35005696 PMC8732863

[20] Crehan C Kesler E Nambiar B Dube Q Lufesi N Giaccone M. The NeoTree application: developing an integrated mHealth solution to improve quality of newborn care and survival in a district hospital in Malawi. BMJ Glob Heal. (2019) 4:860. 10.1136/bmjgh-2018-000860 30713745 PMC6340059

[21] Crehan C Kesler E Chikomoni IA Sun K Dube Q Lakhanpaul M. Admissions to a low resource neonatal unit in Malawi using a mobile App: a digital perinatal outcome audit. JMIR mHealth uHealth. (2020) 8:e16485. 10.2196/16485 33084581 PMC7641784

[22] Gannon H Chimhuya S Chimhini G Neal SR Shaw LP Crehan C. Electronic application to improve management of infections in low-income neonatal units: pilot implementation of the NeoTree beta app in a public sector hospital in Zimbabwe. BMJ Open Qual. (2021) 10:e001043. 10.1136/bmjoq-2020-001043 33472853 PMC7818839

[23] Agile Alliance. Available online at: https://www.agilealliance.org/ (accessed May 3, 2021).

[24] Bangor A Kortum P Miller J. Determining what individual SUS Scores mean: adding an adjective rating scale. J Usability Stud. (2009) 4:114–23.

[25] World Bank. World Bank Data, Under five years and Neonatal mortality trends. (2018). Available online at: https://data.worldbank.org/indicator (accessed July 12, 2018).

[26] Yardley L Morrison L Bradbury K Muller I. The person-based approach to intervention development: application to digital health-related behavior change interventions. J Med Internet Res. (2015) 17:e30. 10.2196/jmir.4055 25639757 PMC4327440

[27] Why You Only Need to Test with 5 Users. Available online at: https://www.nngroup.com/articles/why-you-only-need-to-test-with-5-users/ (accessed September 22, 2020).

[28] What sample size do you really need for UX research? | BetterUX Blog. Available online at: https://www.userzoom.com/blog/what-sample-size-do-you-really-need-for-ux-research/ (accessed September 22, 2020).

[29] Gordon SB Chinula L Chilima B Mwapasa V Dadabhai S Mlombe Y. A Malawi guideline for research study participant remuneration [version 2; referees: 2 approved] (2018). Available online at: 10.12688/wellcomeopenres.14668.1 (accessed September 23, 2020). PMC6329041 30662959

[30] Braun V Clarke V. Using thematic analysis in psychology. Qual Res Psychol. (2006) 3:77–101. 10.1191/1478088706qp063oa

[31] A Quick Introduction to Agile UX Design. Available online at: https://www.webfx.com/blog/web-design/agile-ux-design/ (accessed May 26, 2020).

[32] User Stories and User Story Examples by Mike Cohn. Available online at: https://www.mountaingoatsoftware.com/agile/user-stories [accessed October 6, 2002).

[33] Landis-Lewis Z Kononowech J Scott WJ Hogikyan RV Carpenter JG Periyakoil VS. Designing clinical practice feedback reports: three steps illustrated in Veterans Health Affairs long-term care facilities and programs. Implement Sci. (2020). 15:7. 10.1186/s13012-019-0950-y 31964414 PMC6975062

[34] Ginsburg AS Agyemang CT Ambler G Delarosa J Brunette W Levari S. MPneumonia, an innovation for diagnosing and treating childhood pneumonia in low-resource settings: a feasibility, usability and acceptability study in Ghana. PLoS ONE. (2016) 11:e0165201. 10.1371/journal.pone.0165201 27788179 PMC5082847

[35] Van Leeuwen LM Pronk M Merkus P Goverts ST Anema JR Kramer SE. Barriers to and enablers of the implementation of an ICF-based intake tool in clinical otology and audiology practice—a qualitative pre-implementation study. PLoS ONE. (2018) 13:e0208797. 10.1371/journal.pone.0208797 30533057 PMC6289452

[36] Jedwab RM Hutchinson AM Manias E Calvo RA Dobroff N Glozier N. Nurse motivation, engagement and well-being before an electronic medical record system implementation: a mixed methods study. Int J Environ Res Public Health. (2021) 18:1–23. 10.3390/ijerph18052726 33800307 PMC7967448

[37] León HCM Farris JA. Lean product development research: current state and future directions. Eng Manag J. (2011) 23:29–51. 10.1080/10429247.2011.11431885

[38] GDPR compliance: Meeting EU rules for data privacy. Available online at: https://try.imperva.com/gdprcompliance/?utm_source=google&utm_medium=paidsearch&utm_campaign=jm-fy20q3-nonbrand&utm_content=gs_1771154349_70079615918_349103544887_gdpr&gclid=CjwKCAiA9bmABhBbEiwASb35V8IIdxeuMbdN8lhJD0vIrOK859MlNSxXVAA33YIYiLQWU2fGLKpDDBoCY3oQAvD_BwE (accessed January 25, 2021).

[39] Stevenson AG Tooke L Edwards EM Mangiza M Horn D Heys M. The use of data in resource limited settings to improve quality of care. Semin Fetal Neonatal Med. (2021) 26:101204. 10.1016/j.siny.2021.101204 33579628

[40] Ali SM Giordano R Lakhani S Walker DM. A review of randomized controlled trials of medical record powered clinical decision support system to improve quality of diabetes care. Int J Med Inform. (2016). 87:91–100. 10.1016/j.ijmedinf.2015.12.017 26806716

[41] Harte R Quinlan LR Glynn L Rodríguez-Molinero A Baker PM Scharf T. Human-centered design study: enhancing the usability of a mobile phone app in an integrated falls risk detection system for use by older adult users. JMIR mHealth uHealth. (2017) 5:e71. 10.2196/mhealth.7046 28559227 PMC5470007

[42] Murray D Sedgeworth C Kinnear M Diack L. Evaluation of an electronic paediatric intensive care unit (PICU) medication reconciliation (MR) form. Arch Dis Child. (2016) 101:e2.72–2. 10.1136/archdischild-2016-311535.74 27540258

[43] Crehan C Huq T Kesler E Dube Q Lakhanpaul M Heys M. A qualitative study of healthcare workers perceived barriers and facilitators to neonatal care in a central hospital in Malawi (Poster Abstract 073). Arch Dis Child. (2018) 103:A30. 10.1136/goshabs.73

[44] Shao AF Rambaud-Althaus C Swai N Kahama-Maro J Genton B D'Acremont V. Can smartphones and tablets improve the management of childhood illness in Tanzania? A qualitative study from a primary health care worker's perspective. BMC Health Serv Res. (2015) 15:1–12. 10.1186/s12913-015-0805-4 25890078 PMC4396573

[45] Liese B Blanchet N Dussault G. Background Paper The Human Resource Crisis in Health Services In Sub-Saharan Africa. (2003). Available online at: http://www3.who.int/whosis (accessed November 11, 2020).

[46] Mathauer I Imhoff I. Health worker motivation in Africa: the role of non-financial incentives and human resource management tools. Hum Resour Health. (2006) 4:24. 10.1186/1478-4491-4-24 16939644 PMC1592506

[47] Triplett N Munson S Mbwayo A Mutavi T Weiner B Collins P. Applying human-centered design to maximize acceptability, feasibility, and usability of mobile technology supervision in Kenya: a mixed methods pilot study protocol. Implement Sci Commun. (2021) 2:2. 10.1186/s43058-020-00102-9 33413688 PMC7792108

