# Supplementary material for: Usability-Focused Development and Usage of NeoTree-Beta, an App for Newborn Care in a Low-Resource Neonatal Unit, Malawi
Source: Front Public Health. 2022 Apr 28;10:793314. doi: 10.3389/fpubh.2022.793314 (PMC9096438; doi:10.3389/fpubh.2022.793314)
Supplement: Supplementary file 1 [file Data_Sheet_1.pdf]

Supplementary Material

Table 1 – NeoTree functions and purpose overview
Table 2 – Usage metrics
Table 3 - Demographics, professional & training experience of participants attending app usability
Table 4 – Usability as an Electronic Medical Record (EMR) – data collection feature set
Table 5 – Usability relating to clinical care – clinical care feature set
Table 6 – Usability of the NeoTree Beta app – iterative changes from real-world study
Table 7 – Responses to individual questions of the system usability score
Table 8 – Comparison of findings with previous pilot

Supplementary material 1 – Think aloud usability discussion guide
Supplementary material 2 – SUS score card

Table 1: NeoTree functions and purpose overview

Table 1. NeoTree functions and purpose overview			
**Functions**	**Description of function**	**Aim**	**Goal**
1. Electronic Medical Record (EMR)	Digital data capture at admission, discharge/outcome, and lab data, facilitated via online editor platform.	Improve newborn care	Reduce newborn morbidity & mortality
2. Clinical decision support (CDS)	Facilitated through algorithmic support in emergencies (digital implementation of evidence-based guidelines)		
3. Digital guideline	Clinical management support facilitated through management pages at the end of the app summarising national neonatal guidelines		
4. Education	Educational text and images embedded throughout the app		

Table 2: Usage metrics

Table 2. Usage measures			
**Measure**	**Definition**	**Calculated by**	**Assumptions / notes**
User count	The absolute number of individual health professionals who used NeoTree	• Simple count (n) - Power BI	
Cadre	The proportions of different cadres using NeoTree	• Count of each cadre divided by total users (%) - Power BI	
Coverage	The proportion of patient events recorded by the ward clerk on paper that were captured digitally on NeoTree	• Total number of digital admissions divided by total number of admissions logged by ward-clerk (%) - Excel<br>• Total number of digital outcome exported via NeoTree divided by total number of outcomes (%) - Excel	• Assumes there will be more patients logged on paper than admitted digitally.<br>• Compares totals only - does not match each individual name in the logbook with a digital admission or outcome.
Completion-time	The time taken to complete a digital admission and outcome	• Median number of minutes taken to complete admission (Median (IQR)) - Excel<br>• Median number of minutes taken to complete an outcome (Median (IQR)) - Excel	• Data were exported for the whole 6 months<br>• Only last 2 months of data analysed (to allow for embedding of the intervention).

Table 3: Demographic details and professional and training experience of participants attending think aloud app usability sessions (Age and gender are excluded for confidentiality)

Participant ID	A	B	C	D	E	F	G	H	I	J	K	L
Cadre	NMT	RN	RN	RN	NMT	NMT	NMT	NMT	NO	NMT	RN	RN
Used tablet before?	N	Y	Y	Y	Y	Y	Y	Y	Y	Y	Y	Y
Uses tablet regularly?	N	Y	N	N	N	Y	Y	Y	Y	Y	Y	N
COIN / HBB training?	Y/N	Y/Y	Y/Y	Y/Y	Y/Y	Y/Y	Y/Y	Y/Y	Y/Y	N/N	N/N	Y/Y
Years of experience in newborn care	3	2	2	2	6	5	6	7	5	1	1	7
Attended think-aloud usability interviews	✓	✓	✓	✓	✓	✓	x	x	x	x	x	x
Completed SUS1	✓	✓	✓	✓	✓	✓	✓	✓	x	x	x	x
Completed SUS2	x	✓	x	✓	✓	✓	x	x	✓	✓	✓	✓

NMT = Nurse Midwife Technician, RN = Registered Nurse, , Y = Yes, N = No, COIN = Care of the infant newborn, HBB = Helping Babies Breathe, App = Application, SUS = System Usability Score

Table 4– Usability as an electronic medical record – Data collection feature set

Usability as an Electronic Medical Record – Data Collection Feature Set							
**Overarching Usability Theme**	**Usability themes** with example feedback notes	**Corresponding features** and stories within each feature	**#**	**Ed/ Dev**	**A**	**B**	**Done**
**1. Exhaustiveness of data schema**	**Missing options in dropdowns/MCLs**	**Exhaustive field options**					
	e.g. Microcephaly should be an option in head shape<br>*e.g. Add flucloxacillin to medications list*	All relevant options included in dropdowns/MCLs	5	E	Y	Y	**Y**
	If stillbirth selected include a prompt to fill in info as these babies not usually 'admitted' and we say 'Brought in Dead'	Stillbirth or 'Brought In Dead' (BID) outcome option added	1	E	Y	Y	**Y**
	**Field type prevents complete data entry**	**Appropriate field type for complete data entry**					
	Please allow multiple selections for reason for admission	Reason for admission field changed to multiple choice	1	E	Y	Y	**Y**
	**Missing fields essential for an admission**	**Clinically exhaustive set of fields**					
	Chlorhexidine for umbilicus is needed	Chlorhexidine field added	2	E	Y	Y	**Y**
**2. Prevention of errors to support data integrity**	**Lack of field validation:**	**Field validation**					
	Dashes are hard to find on the keyboard for NeoTree ID - suggest take out dash and stop us entering wrong characters	Field validation added to ID number field on discharge so dash already present & wrong characters cannot be entered	3	D	Y	Y	**Y**
	Date field on discharge form needs to allow dates in the future	Field validation via editor to allow only dates in the future/past	1	D	Y	N	N
	**Contradictory field options:**	**Exclusive field options**					
	Symptom review - crying > normal & Crying < normal needs to be exclusive	Exclusivity option via editor to prevent selection of 2 contradictory MCL options	1	D	Y	N	**N**
**3. Ease of progression through the app**	**Compulsory fields difficult**	**Non-compulsory field to allow progression**					
	Apgar's shouldn't be compulsory as for referrals they are often not available	Apgar field made non-compulsory so HCPs can continue even when apgars are not available	3	E	Y	Y	**Y**
	**Absence of 'Unknown' field option**	**Unknown option to allow progression**					
	Birth History - "Unknown" option for TEO and vit K	included when necessary so HCPs can progress even when information not available	2	E	Y	Y	**Y**
**4. Efficiency of data entry using shortcuts/ calculations**	**Inefficient data entry due to sub-optimal field type**	**Field-types to create shortcuts**					
	Drop down of permanent staff for signature on discharge form	Signature field changed to a dropdown at end of both admission & discharge forms making sign-off quicker	1	E	Y	Y	**Y**
	Follow up date entry on discharge is laborious	Future date field-type to allow selection of future dates from a calendar (so HCPs can schedule follow up clinics	4	D	Y	N	N
	**Lack of simple calculations**	**Advanced field logic:**					
	Age calculation for all babies?	Calculation of age for babies at all ages, including >7 days	1	D	Y	N	N
**5. Navigation of user interface**	**Lack of instructions**	**Calls to action:**					
	Respiratory support page - can you click multiple options?<br>*Feeding page - the multiple selection is not obvious*	Reminder '*Click all that apply*' added to all multiple-choice lists so HCPs know they can enter more than 1 option	5	E	Y	Y	Y
	Apgar field needs "If known"<br>*In modifiable factors - can you put "If present please fill in"*	Caveat messages added where necessary e.g., '*if available*' or '*if present*', or '*only blue fields need completing*' to pages where fields are non-compulsory	2	E	Y	Y	**Y**
	Tap to start on clock not immediately obvious	Instructions 'TAP TO START' made larger in size	1	D	Y	Y	Y
	Tries to tap the page with tasks - consider "when completed tasks continue"	Add instruction to 'tasks' and 'navigation' pages that HCPs only need to click the continue button	1	E	Y	Y	**Y**
	struggled with scrolling<br>*hesitation on scrolling up*	Instructions explaining how to scroll	2	E	Y	N	**N**
	vital signs - number keypad obscures input	Instructions on how to close number keypad	1	E	Y	N	**N**
	**Lack of signposting**	**Signposting**					
	Maternal history - Churches signpost its in alphabetical order	Signpost added to list of churches is in alphabetical order	1	E	Y	Y	**Y**
	Tell user secondary diagnoses can be added later in form on primary discharge page	Add 'secondary diagnoses can be added later' on primary diagnosis page	1	E	Y	Y	**Y**
	Put HW ID instruction in field not page title.	Put example HW-ID in field title rather than on page but doesn't fit in field title, and clearly explained in page content	1	E	Y	N	**N**
	**Confusing layout/design**	**UI design**					
	tried to click writing instead of text box	Make answer boxes immediately obvious / highlighted	2	D	Y	Y	Y
	Confused as lines are grey even for blue fields? can lines be blue?	Make lines for active fields blue consistent with the colour of the writing of active fields	1	D	Y	Y	Y
	Confusion between grey/blue inputs - have a star or something to show next compulsory input	Include some indication on UI which fields are compulsory and which fields are non-compulsory.	3	D	Y	Y	Y
	kept trying to click navigation bar (on a non-click page)<br>*Pages without input fields i.e. navigation pages - different colour?*	Distinguish 'click' pages from 'non-click' pages with different colours	5	D	Y	N	N
	Delete button confused with done button - suggest increase size of done button?	Bigger / brighter continue button	1	D	Y	N	N
**6. Relevancy of content**	**Unnecessary field options:**	**Remove irrelevant field-options**					
	We don't have Intra-nasal / head box oxygen at KCH	Remove intra-nasal & headbox oxygen options from respiratory support field on discharge	1	E	N	Y	**N**

Red font = Outstanding developer adjustments as of Nov 2019, Blue font = outstanding editor adjustments as of Nov 2019. # = number of mentions by participants, Ed = Editor, Dev = Developer, A = criteria A: Aligns with clinical best practice, B = criteria B: practical and feasible to implement within team's capacity.

Table 5 - Usability relating to clinical care – Clinical care feature set

Usability relating to clinical care – Clinical care feature set							
**Overarching Usability theme**	**Usability Themes** with example feedback notes	**Corresponding features** and stories within each feature	**#**	**Ed/ Dev**	**A**	**B**	**Done**
**7. Confidentiality of identifiable information**	**Lack of confidentiality**	**Confidential fields:**					
	Babies name should be confidential on the discharge	All patient identifiable fields in the discharge form made confidential so they are not exported to the database	1	E	Y	Y	**Y**
**8. Cohesion with usual ward process**	**Lack of elements of usual admission process**	**New pages to match ward process:**					
	Can you print management plan?? And include in main app	Overall admission management plan page added to admission form	2	E	Y	Y	**Y**
	Abnormal looking umbilicus - (does that include exomphalos / gastroschisis??)	Exomphalos and gastroschisis management page added	1	E	Y	Y	**Y**
	Health education page at the end of the discharge?	Health promotion for mothers/guardians page added to end of discharge	1	E	Y	Y	**Y**
**9. Embedded educational content**	**Lack of required educational text**	**Educational text:**					
	Can't remember early & late-stage cut-off for sepsis	Clear explanation/reminder of what is early & late neonatal sepsis added to diagnosis at discharge page	1	E	Y	Y	Y
	**Lack of required educational images**	**Educational images:**					
	Method of checking the tone should be reinforced	Pictures added re how to measure tone on admission	1	E	Y	Y	**Y**
**10. Locally coherent language**	**Locally inappropriate language**	**Locally understandable language**					
	Birth History - "Vit K given at birth"<br>*light palpation of abdomen rather than softly*<br>*< or > not understood on 18 hrs*<br>*Still birth - leave in form*	e.g. Specify vit K 'given at birth'<br>e.g. Change from 'softly' to 'lightly' palpate the abdomen<br>e.g. Remove > or < symbols - write out<br>e.g. Discuss nomenclature for still births & BIDs with team	5	E	Y	Y	**Y**
	Adjust maturity score to say Ballard??<br>*No one is using the coin maturity score*	Change 'maturity score' to 'Ballard score'	3	E	N	Y	**N**
	FeFo write out - as iron and folate - some HCWs who are not midwives by training don't know this	Write out 'FeFo' (Ferrous sulphate &Folate)	3	E	Y	Y	**Y**
**11. Adaptability of UI according to resources**	**Lack of resources/ confidence using resources required to complete app**	**Configuration page**					
	Users probably happy with stet for lungs but not heart<br>*Not confident with Stethoscope - needs training*<br>*Tape measures may not be available*	Add configuration options to editor so the app can be tailored to availability of resources e.g. Stethoscopes, Tape measures, by the nurse in charge.	3	D/ E	Y	Y	**Y**
**12. Print-out design to facilitate handover**	**Printout heading confusing**	**Clear printout headings**					
	Double heading of diagnosis on admission print out.	adjusted on print out to facilitate easy hand over process e.g. remove extra diagnosis section heading	1	E	Y	Y	**Y**
	**Difficult to see abnormal data on printout**	**Data highlighting on printout:**					
	highlight abnormalities on the printout - editor needs bold/colour capability<br>*Highlight BIRTH DATE not admission date on printout*<br>*Highlight data on admission that needs to be entered into Discharge form*	Highlight 'abnormal' data / important fields on the print-out to facilitate easy handover	3	D	Y	N	N

Red font = Outstanding developer adjustments as of Nov 2019, Blue font = outstanding editor adjustments as of Nov 2019. # = number of mentions by participants, Ed = Editor, Dev = Developer, A = criteria A: Aligns with clinical best practice, B = criteria B: practical and feasible to implement within team's capacity

Table 6– Usability of the NeoTree beta app – iterative changes made during real-world pilot

Usability themes findings - generated iteratively during first 6 months of rollout							
**Usability – data collection feature set**							
**Overarching Usability Theme**	**Usability Themes** with example feedback notes	**Corresponding features** and stories within each feature	**#**	**Ed/ Dev**	**A**	**B**	**Done?**
**1. Exhaustiveness of data schema**	**Missing options in dropdowns/MCLs**	**Exhaustive field-options**					
	Please include option for 4xSP doses in antenatal section	All relevant option included in MCLs & Dropdowns	1	E	Y	Y	**Y**
	Please include option for Respiratory distress of the newborn (term) in diagnosis list		1	E	Y	Y	**Y**
	Unrecordable option for BS??		1	D	Y	N	**N**
	Cardiac clinic on Thursdays as follow up option?		1	E	Y	Y	**Y**
	Congenital abnormalities should include Hydrocephalus, Spinal deformity, cleft lip & palate, ano-rectal malformation		1	E	Y	Y	**Y**
	Outcomes on the discharge should include discharged, Absconded, transferred to other ward, transferred to other hospital, NND<24 hrs and NND > 24 hrs		1	E	Y	Y	**Y**
	**Missing fields essential for newborn admission**	**Clinically exhaustive set of fields:**					
	Include examination of the palate in examination section	Palate field added so HCPs can document examination of palate	1	E	Y	Y	**Y**
	Please include no. of sibling's dead field as this was on old MOH form	Number of sibling's dead field not included (as removed previously)	1	E	N	N	**N**
	BID should have a focused Hx and exam	New fields added to BID/Stillbirth script	1	E	Y	Y	**Y**
	Can you include a cause of BID/Stillbirth field?		1	E	Y	Y	**Y**
	Can you include modifiable factors for babies BID/Stillbirth?		1	E	Y	Y	**Y**
**3. Ease of progression through app**	**Compulsory fields sometimes difficult**	**Non-compulsory fields:**					
	Can you grey out the patient 1st name and surname for BID/Stillbirths?	Make name fields optional or greyed out completely for BIDs	1	E	Y	Y	**Y**
**5. Navigation of UI**	**Lack of instructions**	**Calls to action:**					
	How do we enter the name of dumped baby when they have no name?	Instructions included on how to name a dumped baby	1	E	Y	Y	Y
**Usability - clinical decision support feature set**							
**9. Embedded education & decision support**	**Lack of educational text**	**Educational text:**					
	Good to show basic comparison table on surgical Mx page	Table added to surgical gastroschisis / exomphalos page indicating how to distinguish between the two diagnoses	1	E	Y	Y	Y
	Include management of gastroschisis vs exomphalos	Management page for gastroschisis/ exomphalos revised after surgical review	1	E	Y	Y	**Y**
	**Lack of educational images**	**Educational images:**					
	We need a picture of 'strong distal flexion' in Thompson score	Picture of strong distal flexion	1	E	Y	N	N
	Picture of weighing baby naked would remind us to weigh baby naked!	Picture of weighing baby naked	1	E	Y	N	N
	Picture of how to do measure OFC properly would be helpful	Picture of measuring OFC	1	E	Y	N	N

Table 7 – SUS responses and scores

Responses to SUS		
Questions are answered using a likert scale of 1-5 where 1 = strongly disagree and 5 = strongly agree	**SUS1** (After 1 use of NeoTree App, pre app usability adjustments)	**SUS2** (After 6 months of NeoTree use, post app usability adjustments)
**Question**	**Mean (SD)**	**Mean (SD)**
1. I think I would like to use this system frequently	4.8 (0.5)	4.8 (0.5)
2. I found the system unnecessarily complex	1.5 (1.1)	1.3 (0.5)
3. I thought the system was easy to use	4.8 (0.5)	4.8 (0.5)
4. I think that I would need the support of a technical person to be able to use this system	1.3 (0.5)	1.4 (0.7)
5. I found the various functions in this system were well integrated	4.6 (0.5)	4.6 (0.7)
6. I thought there was too much inconsistency in this system	2.0 (0.5)	1.3 (0.5)
7. I would imagine that most people would learn to use this system	4.4 (0.5)	4.1 (1.5)
8. I found the system very cumbersome to use	1.9 (0.5)	1.9 (1.0)
9. I felt very confident using the system	5.0 (0.5)	4.9 (0.4)
10. I needed to learn a lot of things before I could get going with this system	1.6 (0.5)	1.6 (0.7)
Overall SUS score*	**88.1** (10.2)	**89.4** (7.0)

SUS = System Usability Score, IQR = Interquartile range, SD = Standard Deviation, *Overall mean SUS score calculated by subtracting 1 from odd numbered question scores, subtracting even numbered question scores from 5 and then adding them up and dividing the total by 10.

Table 8 – comparison of findings with previous pilot

Comparison of findings with previous pilot study		
	**Previous pilot (2017)**	**This study (2019)**

Aim	To develop NeoTree from x --> y	Alpha prototype --> MVP1	MVP1 --> MVP2
Method	**Setting**	Zomba Central Hospital (ZCH)<br>District level hospital - Southern Region of Malawi, permanent neonatal staff = 20	Kamuzu Central Hospital (KCH) - Lilongwe<br>Tertiary referral centre - Central Region of Malawi, permanent neonatal staff = 10
	**Procedure:** Think aloud interviews (n)	13	6
	**Procedure:** Real-world pilot	1 month of use phased in over time	6 months using NeoTree as part of usual care
		NeoTree completed in addition to paper, on a temporary basis - for duration of study only	NeoTree completely replaced paper,<br>on a permanent basis<br>policy to use NeoTree on all neonates
		No technical support in place<br>Author on site 9-5 Mon-Fri	3x NeoTree Ambassadors present<br>Author on site for 1st & last month only
Results	Total participants (n)	43	93
	Qualitative usability themes (n)	11	12
	Qualitative usability theme names	*1. Type of question*<br>*2. Sequence of fields*<br>*3. Language*<br>*4. Completing fields*<br>*5. Using timer*<br>*6. Understanding instructions*<br>*7. Length of question*<br>*8. Information not available*<br>*9. Proceeding through the app*<br>*10. Navigation*<br>*11. Drop-down menus*	Themes relating to data capture:<br>*1. Exhaustiveness of data schema*<br>*2. Prevention of errors to support data integrity*<br>*3. Ease of progression through the app*<br>*4. Efficiency of data entry using shortcuts/calculations*<br>*5. Navigation of user interface*<br>*6. Relevancy of content*<br>Themes relating to clinical care:<br>*7. Confidentiality of identifiable information*<br>*8. Cohesion with usual ward process*<br>*9. Embedded educational content*<br>*10. Locally coherent clinical language*<br>*11. Adaptability of UI according to resources*<br>*12. Print-out design to facilitate handover*
	SUS	80.8 --> 86.1 (n=13,13)	88.1 --> 89.4 (n=8,8)
	NeoTree admissions captured (n)	134	1323
	NeoTree outcomes captured (n)	129	1197
	Coverage of actual admissions (%)	70	100
	Completion time - admissions (mins)	Mean = 37 (range 18-59)	Median = 16 (IQR 11, 21)
	Completion time - outcomes (mins)	n/a - (completed by the authors)	Median = 8 (IQR 5, 12)
	User cadre	Mainly nursing cadres (53% students)	Mainly nursing cadres (22% students)

Supplementary Material 1 - Think aloud Usability Discussion guide: Neutral prompts for App usability interviews

Introduction

Hi, thank you for your time today. We would like to use your first use of the NeoTree as a way of exploring the usability of the NeoTree application and to understand your satisfaction of your experience using it for the first time. Don't worry it is not a test. We would just like you to work your way through the application on this tablet and see if it makes sense. Does that sound ok?

We just want to make clear right away that you can't do anything wrong today. We just want to find out more about whether the app works for you and what might be useful so that we can improve it. We are very interested in your open and honest feedback, negative, neutral or positive. Any feedback at all is welcome. Does that make sense?

So when you use the app for the first time, try to think out loud as much as possible: say what you're looking at, what you're trying to do, and what you're thinking.

With your permission, we're going to record what happens on the screen, your face and our conversation. The recording will only be used by people working on this project. And it helps us, because we don't have to take as many notes. Try to read out loud as your progress through the app and explain what you are doing as you go, we will prompt you. Does that all sound ok?

Please let us reiterate that you are under no obligation to participate and are free to leave now or at any time if you do not wish to continue. Do you have any questions? Is there anything you don't understand?

Tasks

I am going to ask you to complete a new admission to the nursery using the Neotree app and then a discharge form. I will take you through the different sections of the app. Remember to keep on thinking out loud as you complete each section. If possible, talk me through each thing I show you and tell me if you think it makes sense and could be useful to you. And remember I just want you to **think out loud as much as possible.**

Prompts: Facilitator to use the following neutral prompts:

- What are you looking at?
- What are you trying to do?
- What are you thinking?
- Why did you click that?
- What would you do next?
- Is this clear or un-clear? In what way?
- Is this useful or interesting to you? In what way?
- What else is missing?
- The 5 whys – why x 5 e.g. why did you press that? Why did you think that? Why do you need this? Why do you want that? Why did you hesitate just then? …

Follow up questions (optional if there is time:

How do you feel the tablet will affect your interaction with the baby or the mother?

Overall, how did the experience of using the NeoTree app to admit a baby compare with writing it down / filling in a written admission form

Supplementary material 2 – SUS score card

		Strongly Disagree				Strongly Agree
1.	I think that I would like to use this system frequently.<br>***Ndikuganiza kuti ndikhoza kumagwiritsa ntchito dongosolo la NeoTree pafupipafupi.***	1	2	3	4	5
2.	I found the system unnecessarily complex.<br>***Ndikuona ngati dongosolo la NeoTree angofuna kulipanga kukhala lovuta.***	1	2	3	4	5
3.	I thought the system was easy to use.<br>***Dongosolo la NeoTree ndi losavuta kugwiritsa ntchito.***	1	2	3	4	5
4.	I think that I would need the support of a technical person to be able to use this system.<br>***Ndikuganiza kuti ndingafune munthu wodziwa za kompyuta kundithandiza kuti ndigwiritse bwino ntchito dongosolo la NeoTree.***	1	2	3	4	5
5.	I found the various functions in this system were well integrated.<br>***Ndaona kuti zichitochito za mu dongosolo la NeoTree ndizolumikizana bwino.***	1	2	3	4	5
6.	I thought there was too much inconsistency in this system.<br>***Ndikuona kuti muli zinthu zambiri zosalumikizana mu dongosolo la NeoTree.***	1	2	3	4	5
7.	I would imagine that most people would learn to use this system very quickly.<br>***Ndikulingalira kuti anthu ambiri aphunzira mwamsanga kugwiritsa ntchito dongosolo la NeoTree.***	1	2	3	4	5

8. I found the system very cumbersome to use.
- ***Dongosolo la NeoTree lachulukisa zochita choncho likukhala lovuta kugwiritsa ntchito.***

1	2	3	4	5

9. I felt very confident using the system.
Sindikuzikayikira konse pogwiritsa ntchito dongosolo la NeoTree.

1	2	3	4	5

10. I needed to learn a lot of things before I could get going with this system.

Ndinafunika kuphunzira zinthu zambiri ndisanayambe kugwiritsa ntchito dongosolo la NeoTree.

1	2	3	4	5
